# Supplementary material for: Rhamnopyranoside-Based Fatty Acid Esters as Antimicrobials: Synthesis, Spectral Characterization, PASS, Antimicrobial, and Molecular Docking Studies
Source: Molecules. 2023 Jan 18;28(3):986. doi: 10.3390/molecules28030986 (PMC9919056; doi:10.3390/molecules28030986)
Supplement: Supplementary file 1 [file molecules-28-00986-s001.zip › molecules-2154091-supplementary.pdf]

Supplementary Information (SI)

Rhamnopyranoside-Based Fatty acid Esters as Antimicrobials: Synthesis, Spectral Characterization, PASS, Antimicrobial, and Molecular Docking Studies

Abul Fazal Muhammad Sanaullah [1,†], Puja Devi [1,†], Takbir Hossain [1,†], Sulaiman Bin Sultan [1,†], Mohammad Mohib Ullah Badhon [1,†], Md. Emdad Hossain [2,†], Jamal Uddin[3,†], Md. Abdul Majed Patwary[4,†], Mohsin Kazi[5,*], [†], and Mohammed Mahbubul Matin [1, †,*]

Table of contents

Sl. No.	Name of spectrum	Compound	Page
1.	FT-IR spectra	**4, 6,7, 9, 11**	1, 8, 10, 15, 20
2.	^{1}H NMR spectra (with expansion)	**4, 5, 6, 7, 8, 9, 10, 11**	2, 3, 5, 6, 8, 9, 11, 12, 13, 15,16,17,18, 21
3.	^{13}C NMR spectra (with expansion)	**4, 5, 6, 7, 8, 9, 10**	3, 7, 10, 12, 14, 17, 19
4.	2D DEPT	**4**	4
5.	2D COSY spectra	**4, 8, 10**	4, 14, 19
6.	2D HSQC spectra	**10**	20
7.	2D HMBC spectra	**4, 5**	5, 7

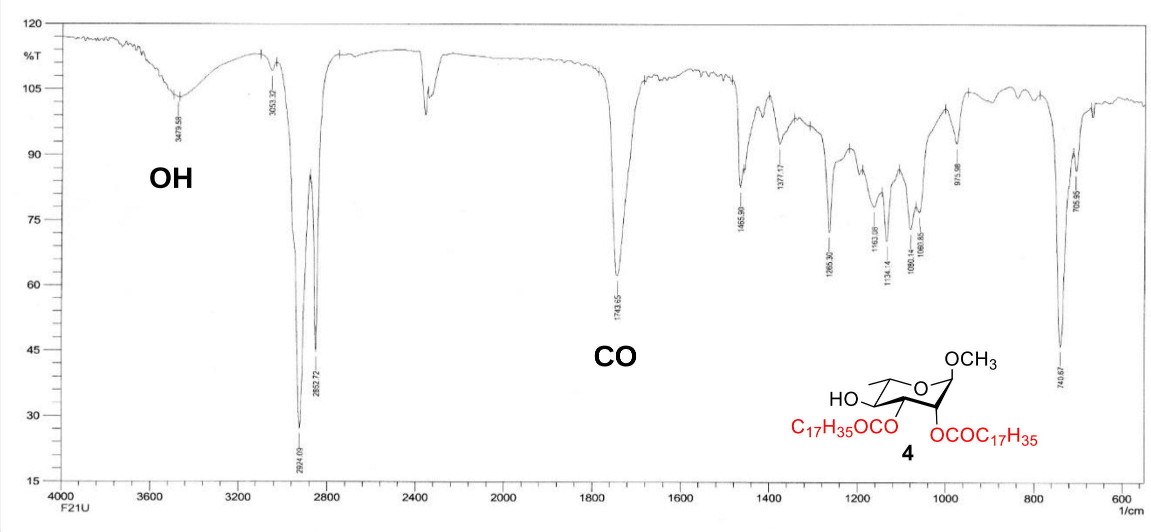

Figure S1. FT-IR (neat) spectrum of compound **4**.

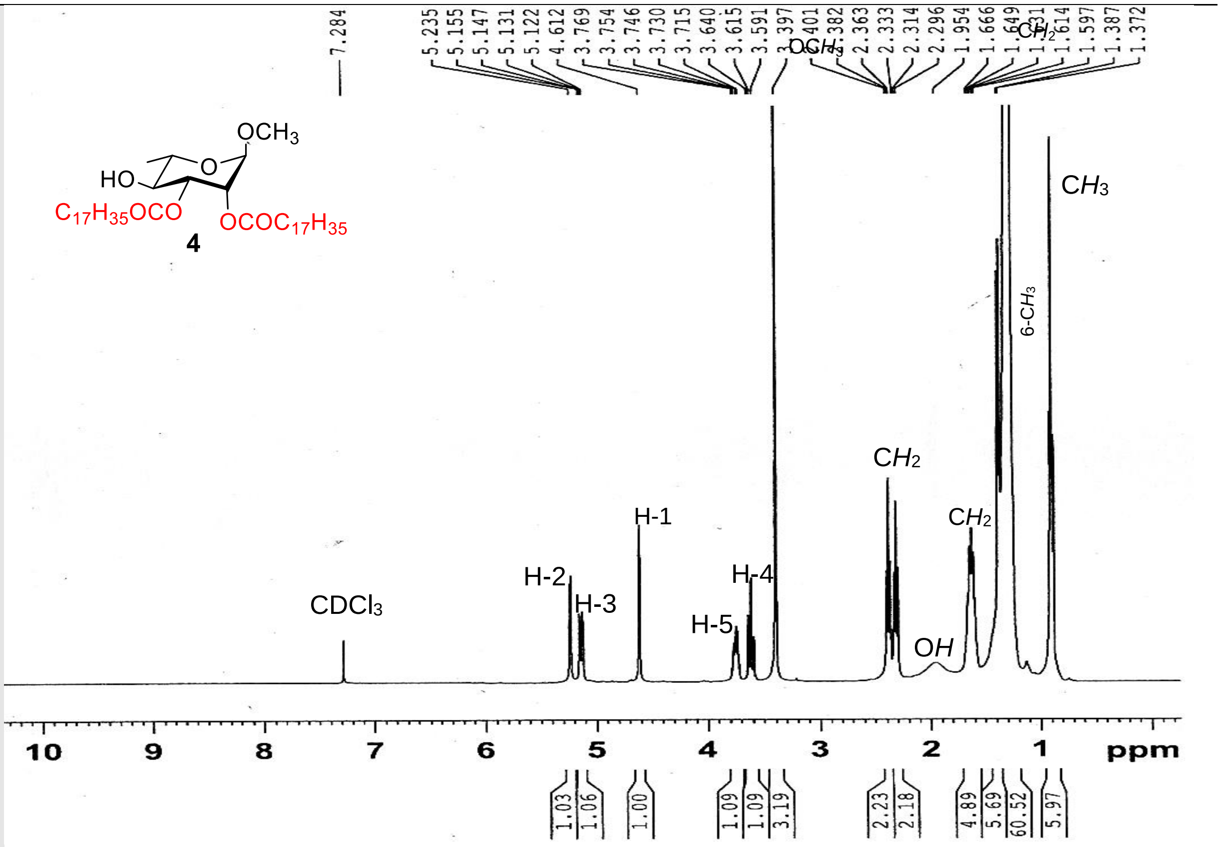

Figure S2. ^{1}H NMR (400 MHz, $CDCl_3$) spectrum of compound **4.**

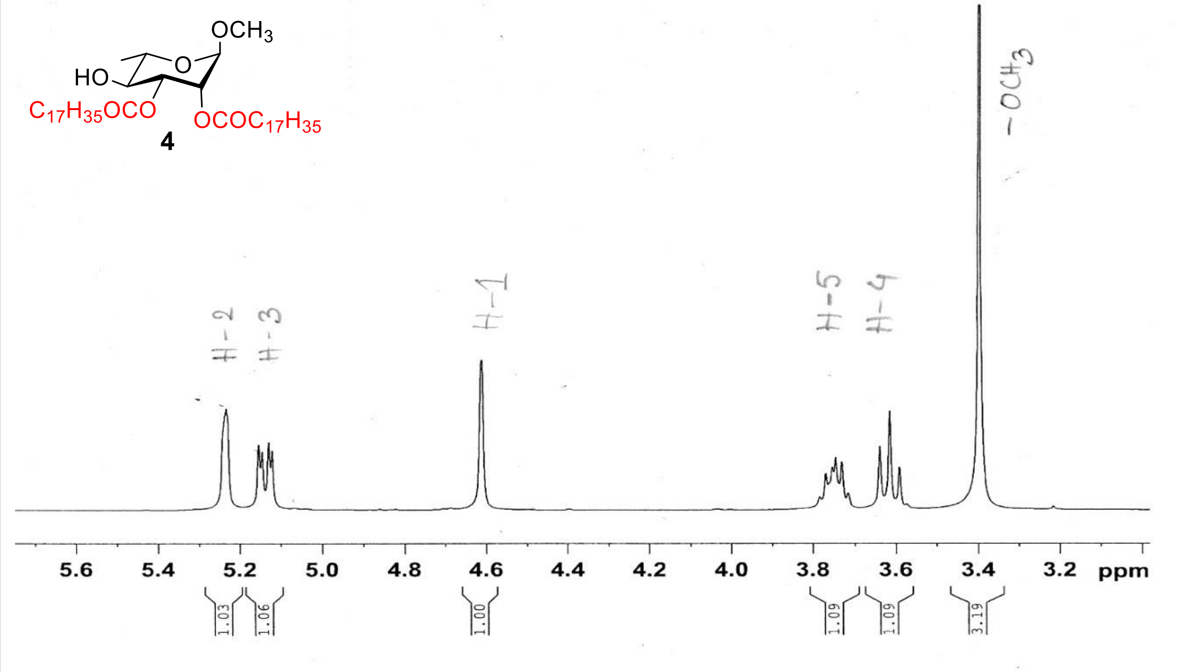

Figure S3. ^{1}H NMR (400 MHz, $CDCl_3$) spectrum of compound **4** (expansion).

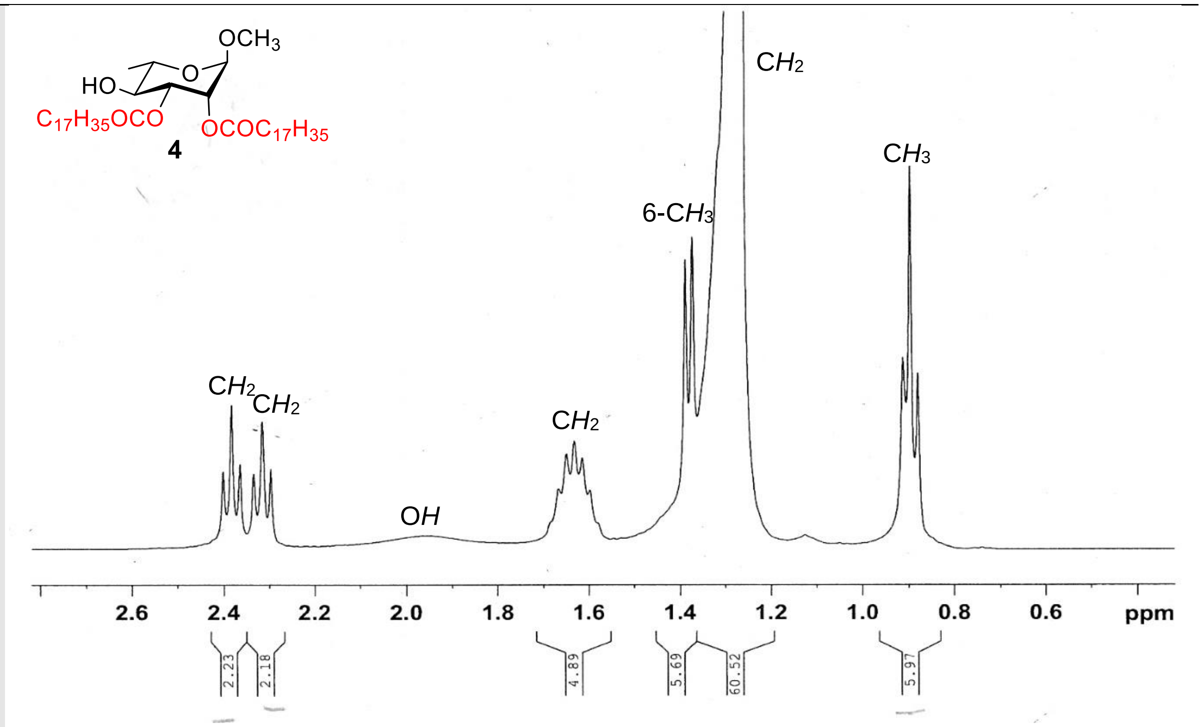

Figure S4. ^{1}H NMR (400 MHz, $CDCl_3$) spectrum of compound **4** (expansion).

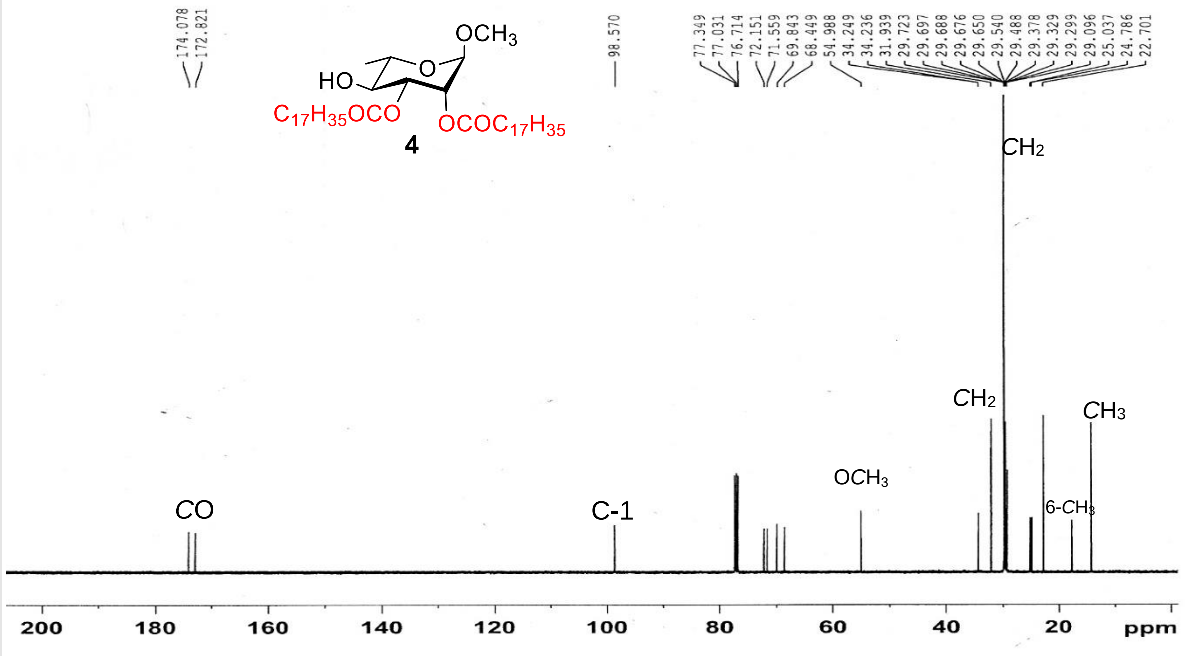

Figure S5. ^{13}C NMR (100 MHz, $CDCl_3$) spectrum of compound **4**.

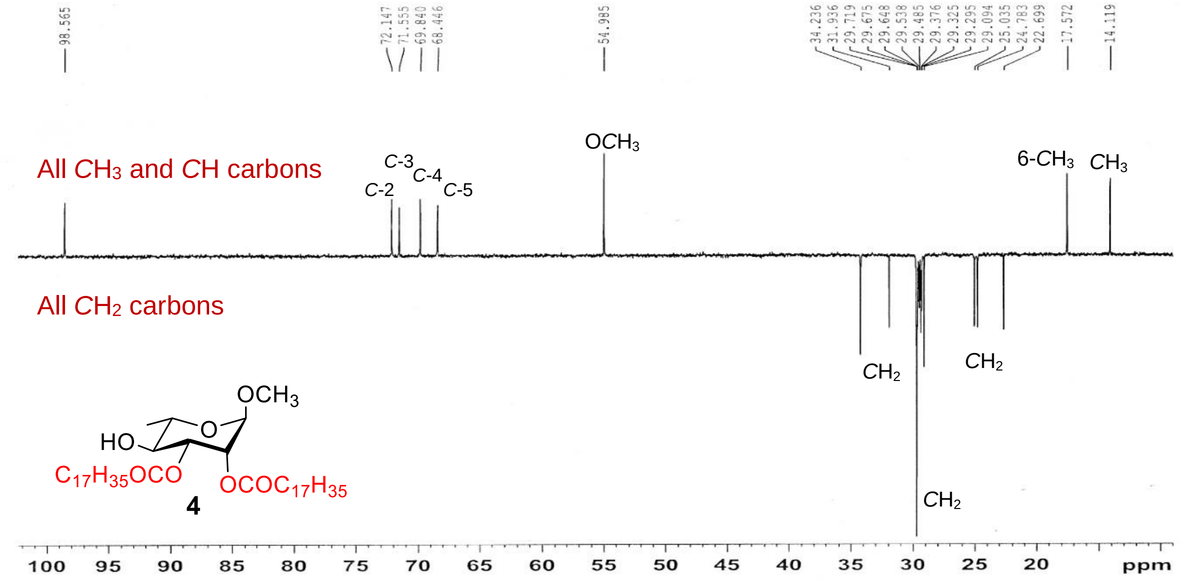

Figure S6. DEPT-135 NMR spectrum of compound **4**.

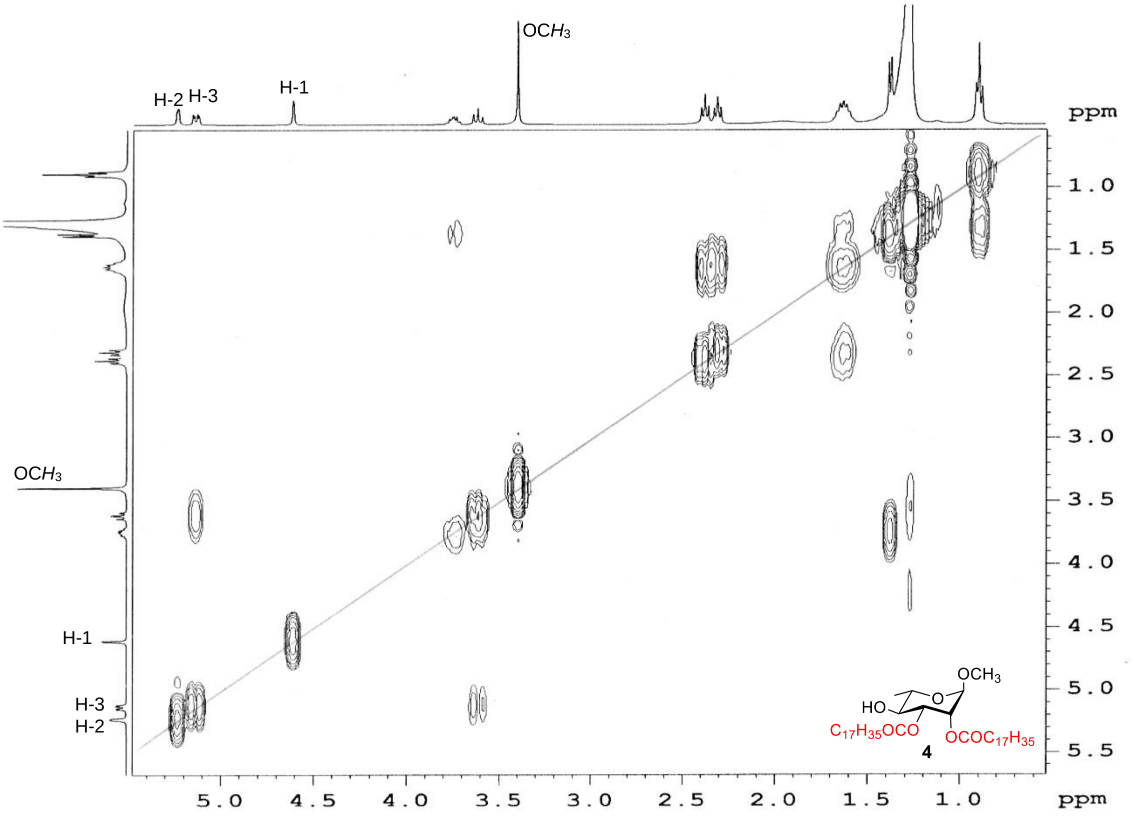

Figure S7. 2D COSY spectrum of compound **4**.

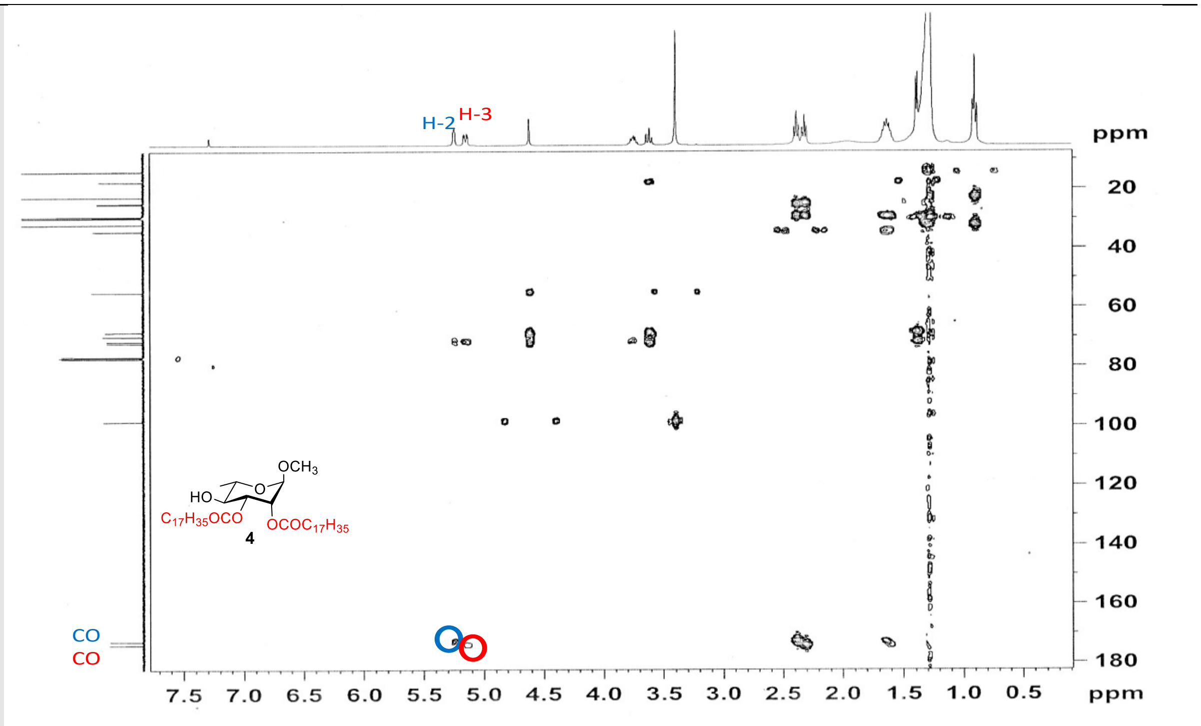

Figure S8. 2D HMBC spectrum of compound **4**.

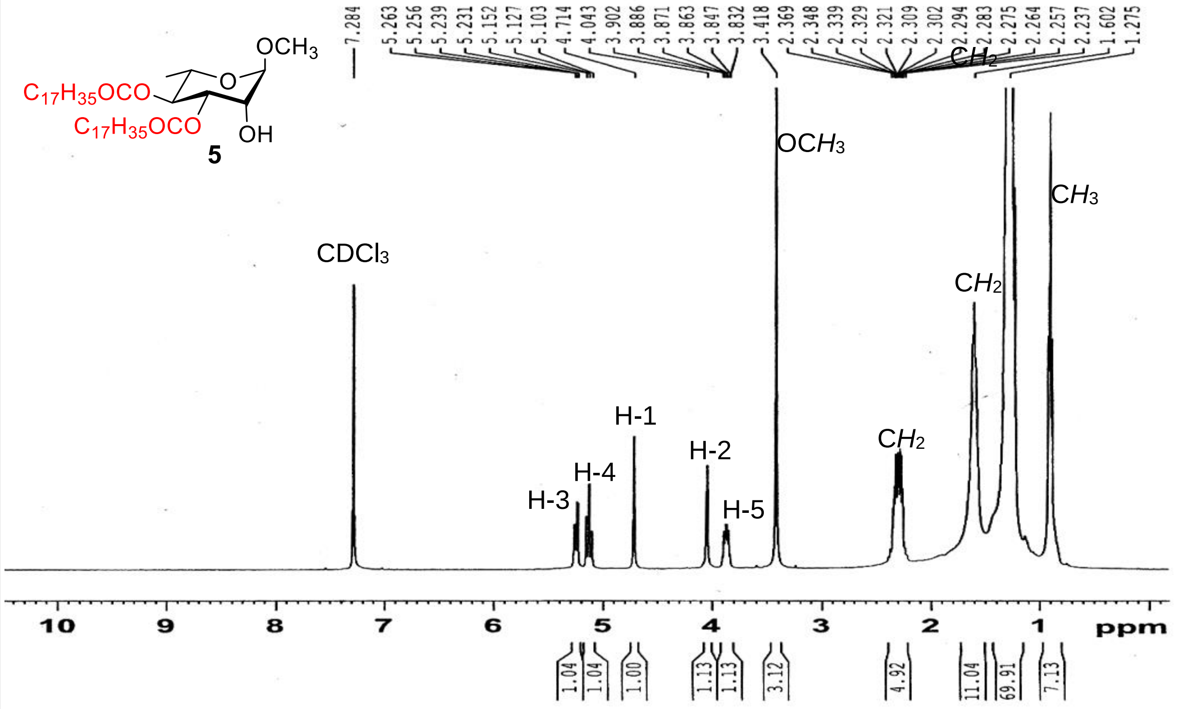

Figure S9. ^{1}H NMR (400 MHz, $CDCl_3$) spectrum of compound **5**.

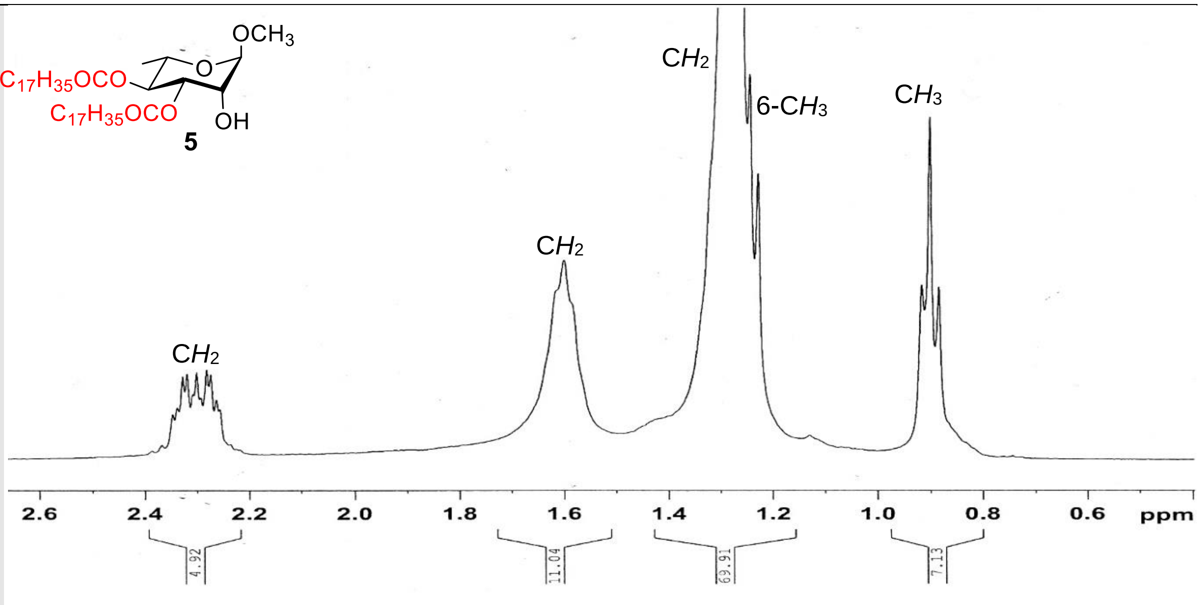

Figure S10. ^{1}H NMR (400 MHz, $CDCl_3$) spectrum (Expansion) of compound **5**.

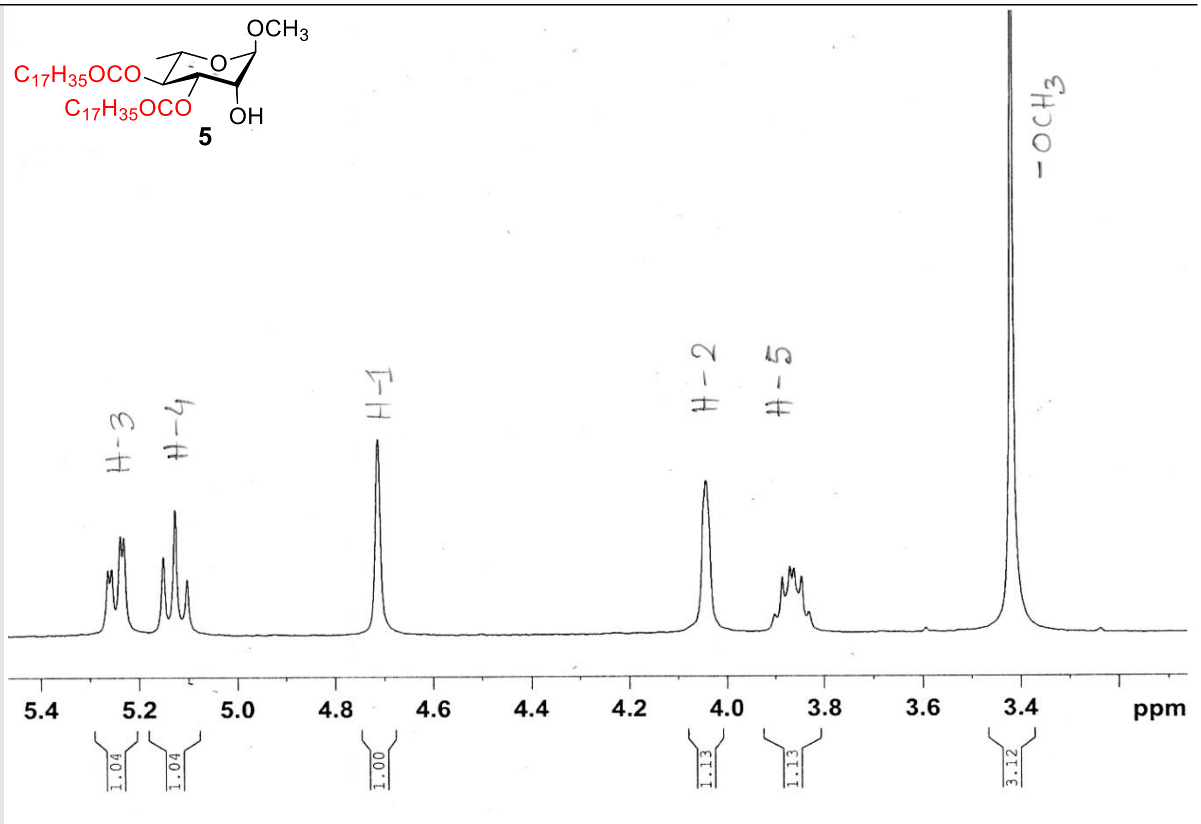

Figure S11. ^{1}H NMR (400 MHz, $CDCl_3$) spectrum (Expansion) of compound **5**.

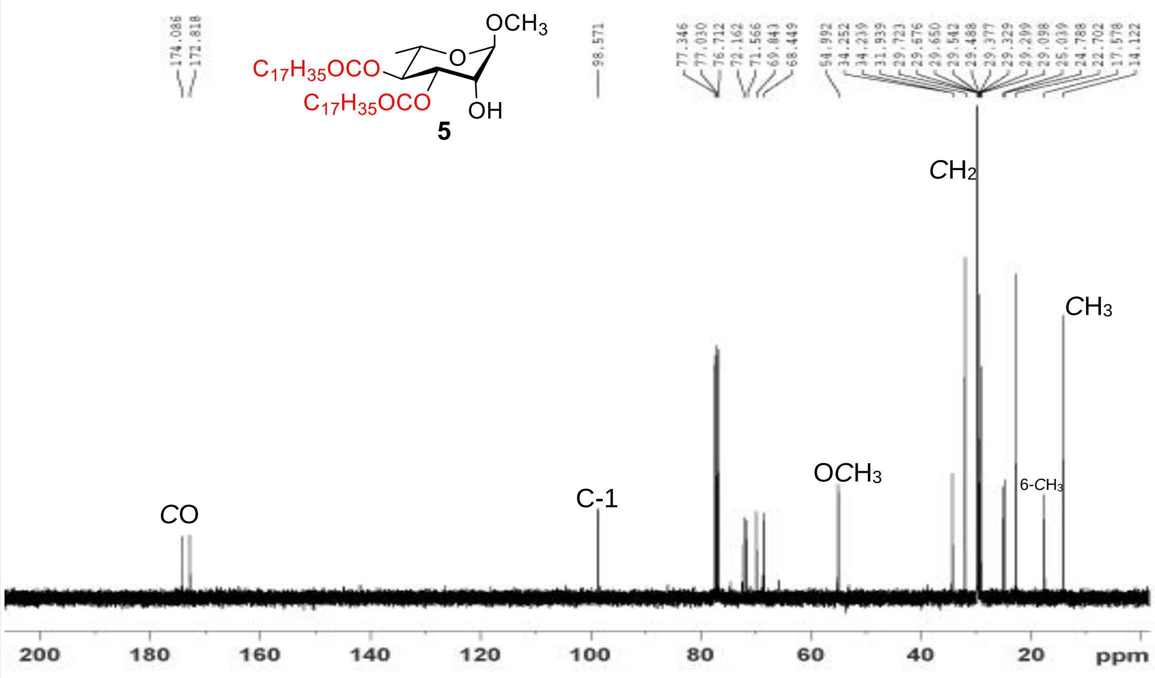

Figure S12. ^{13}C NMR (100 MHz, $CDCl_3$) spectrum of compound **5**.

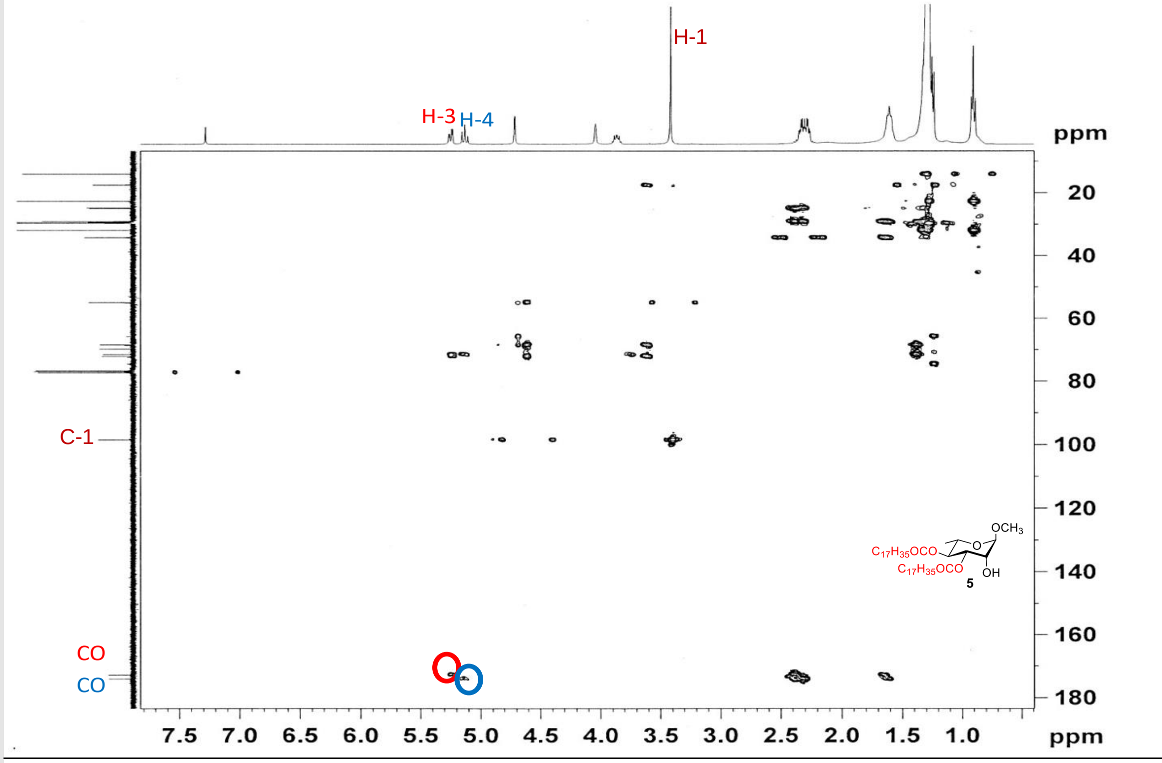

Figure S13. 2D HMBC spectrum of compound **5**.

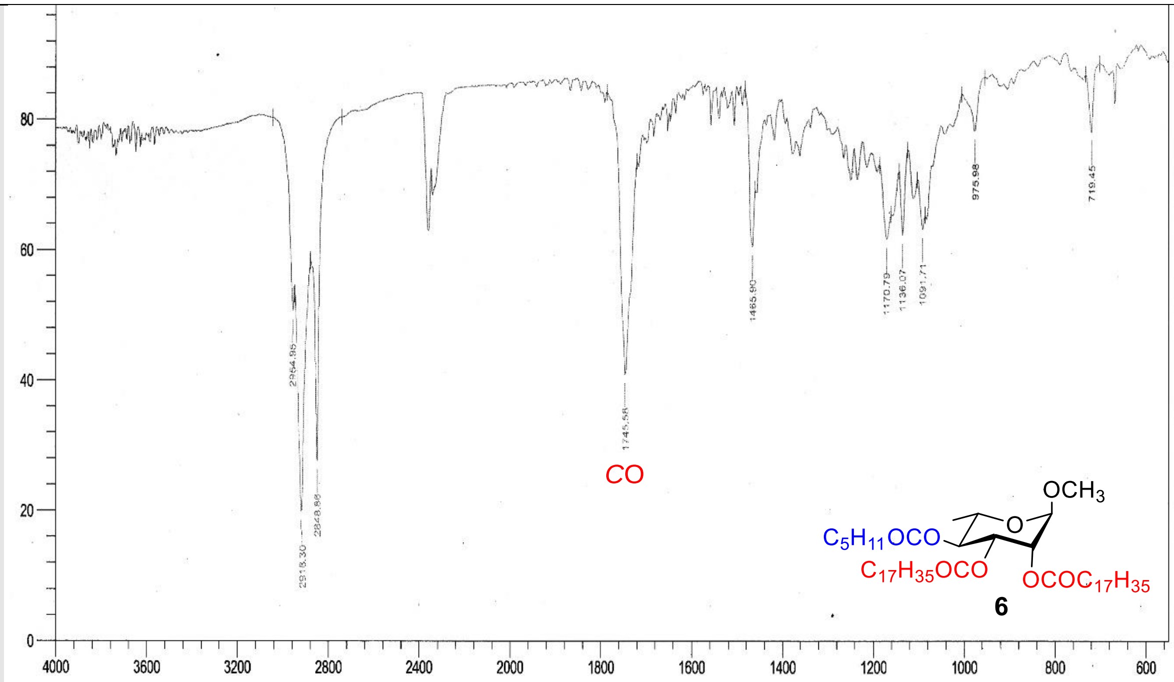

Figure S14. IR (neat) spectrum of compound **6**.

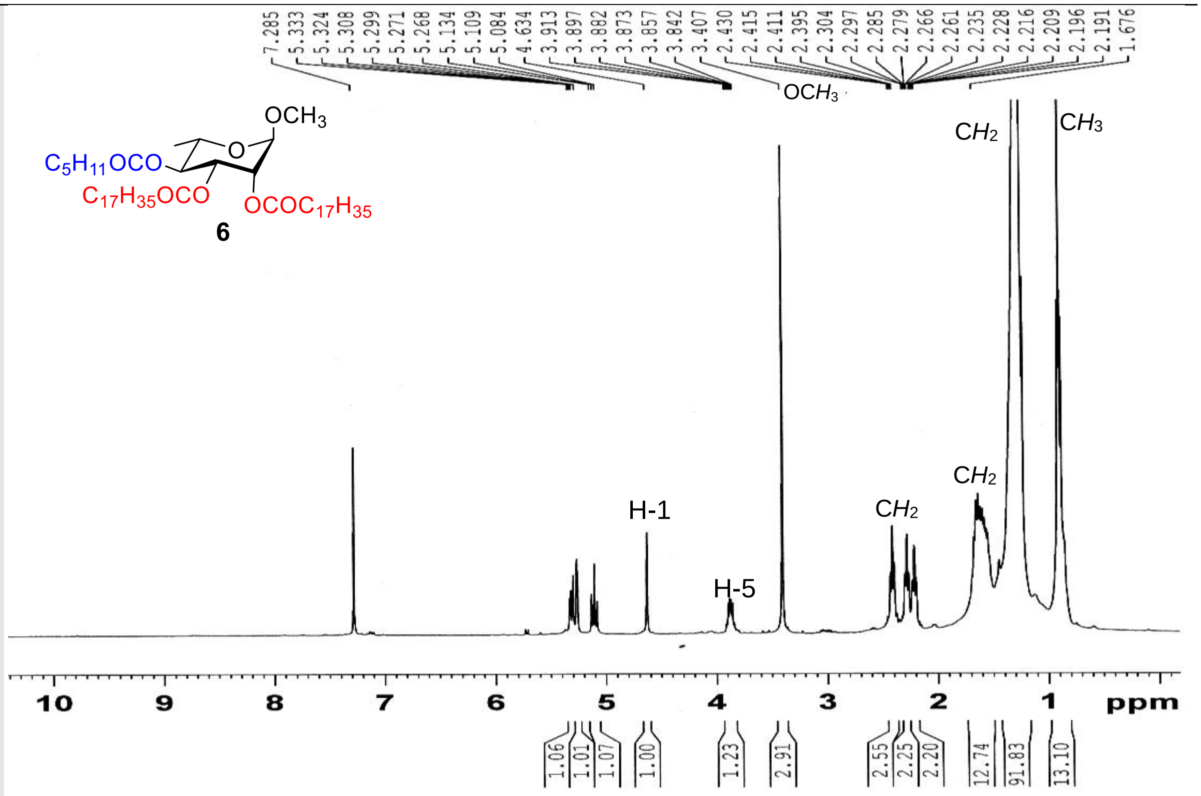

Figure S15. ^{1}H NMR (400 MHz, $CDCl_3$) spectrum of compound **6**.

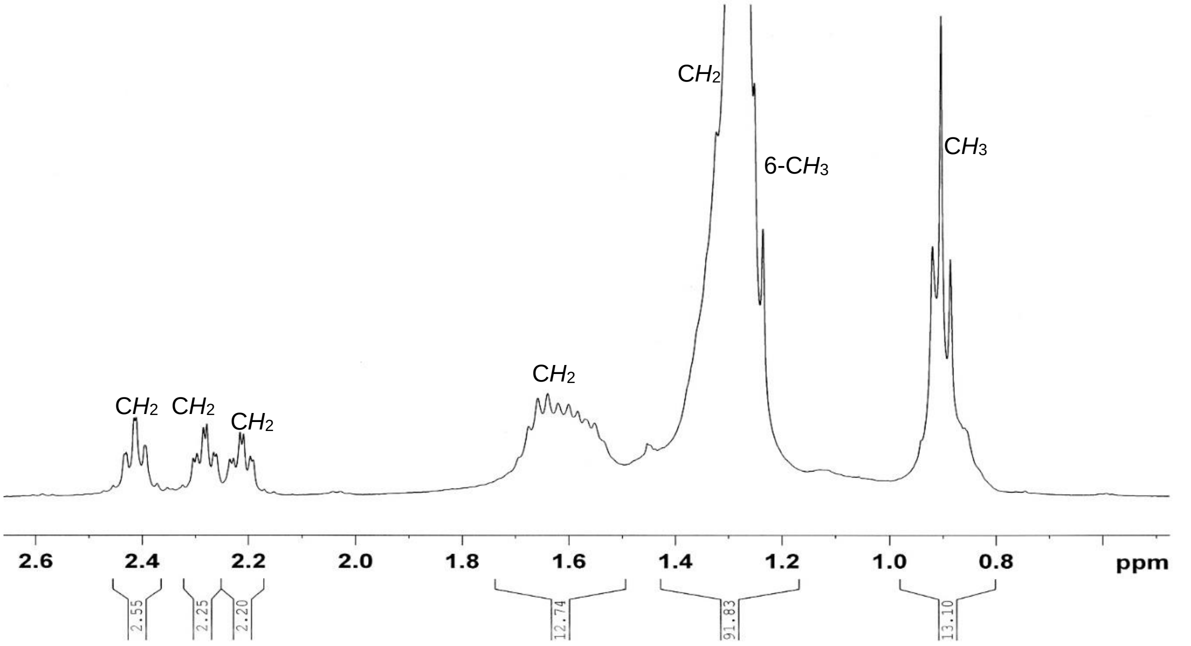

Figure S16. ^{1}H NMR (400 MHz, $CDCl_3$) spectrum (Expansion) of compound **6**.

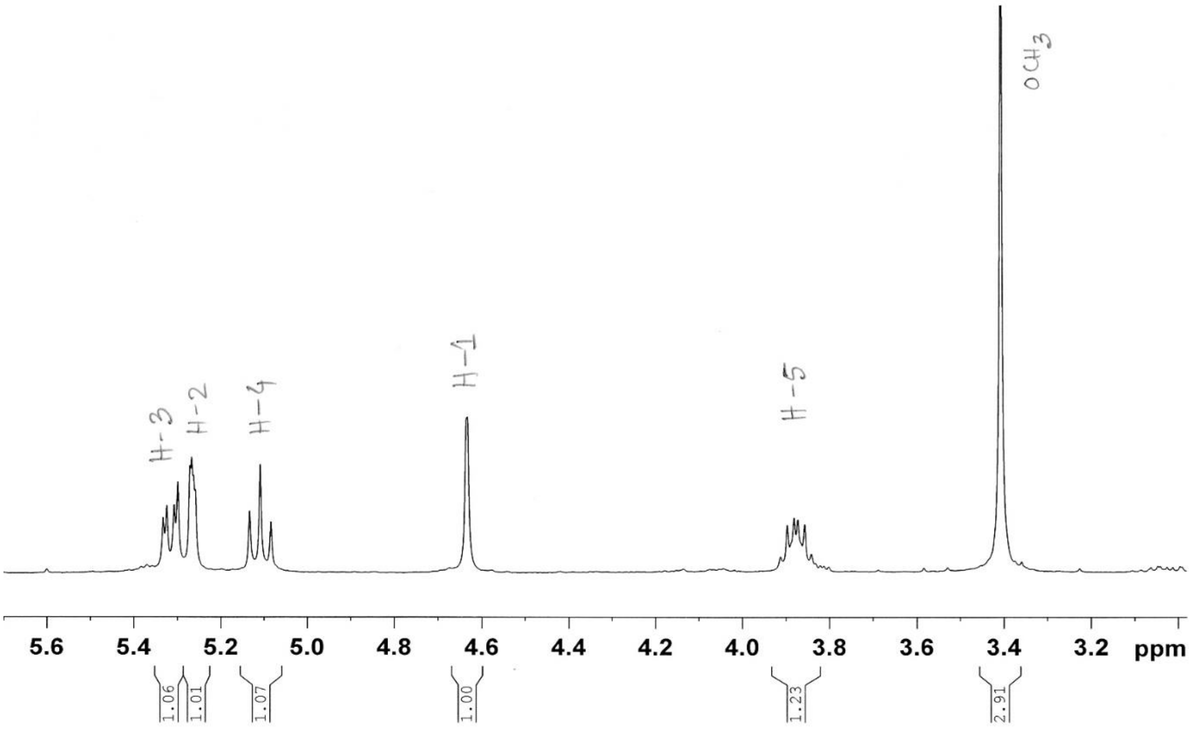

Figure S17. ^{1}H NMR (400 MHz, $CDCl_3$) spectrum (Expansion) of compound **6**.

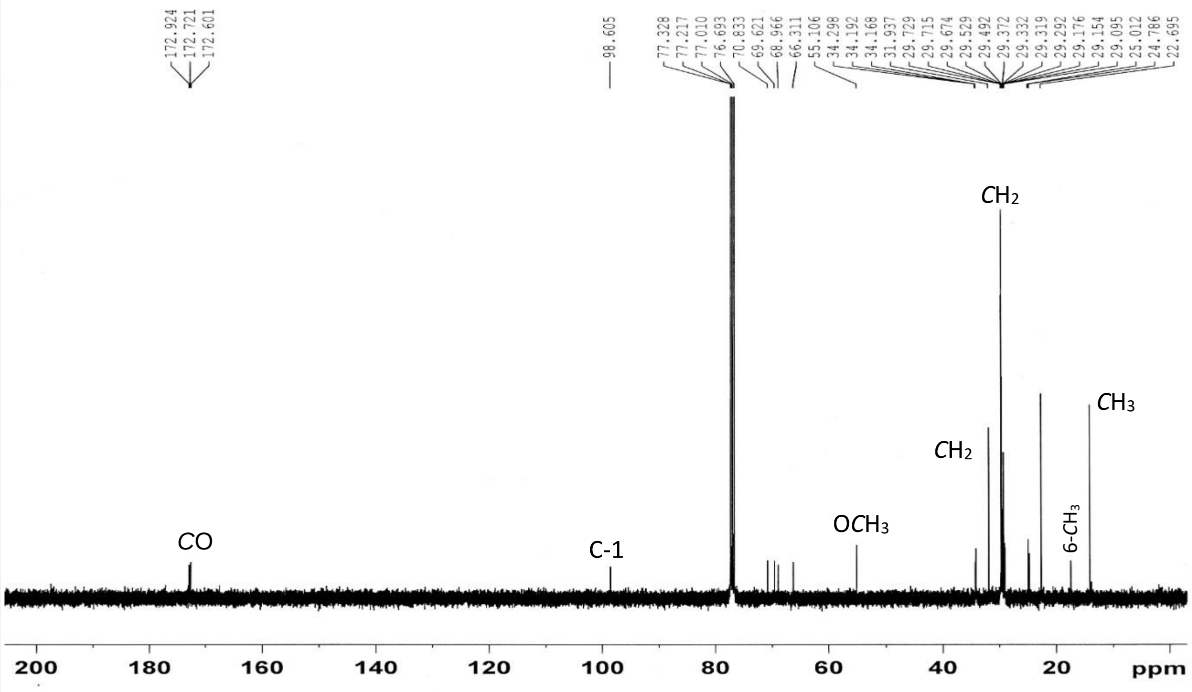

Figure S18. ^{13}C NMR (100 MHz, $CDCl_3$) spectrum of compound **6**.

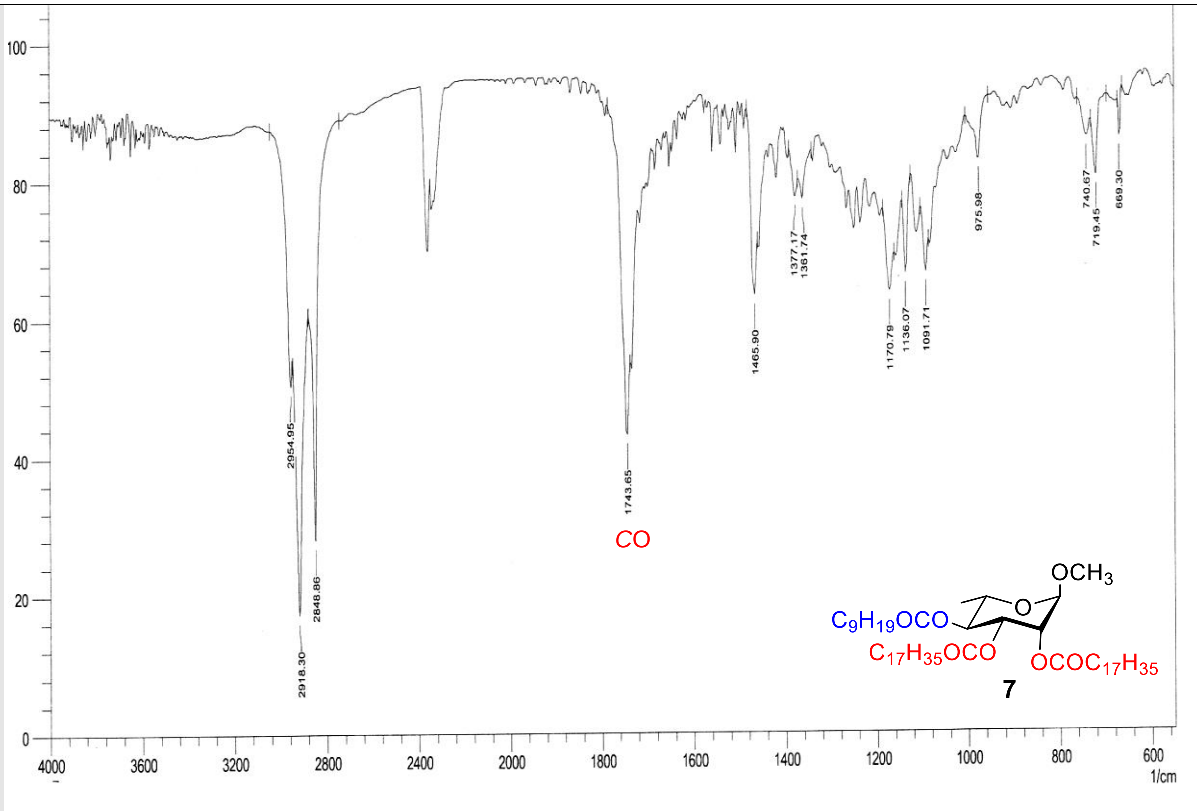

Figure S19. IR (neat) spectrum of compound **7**.

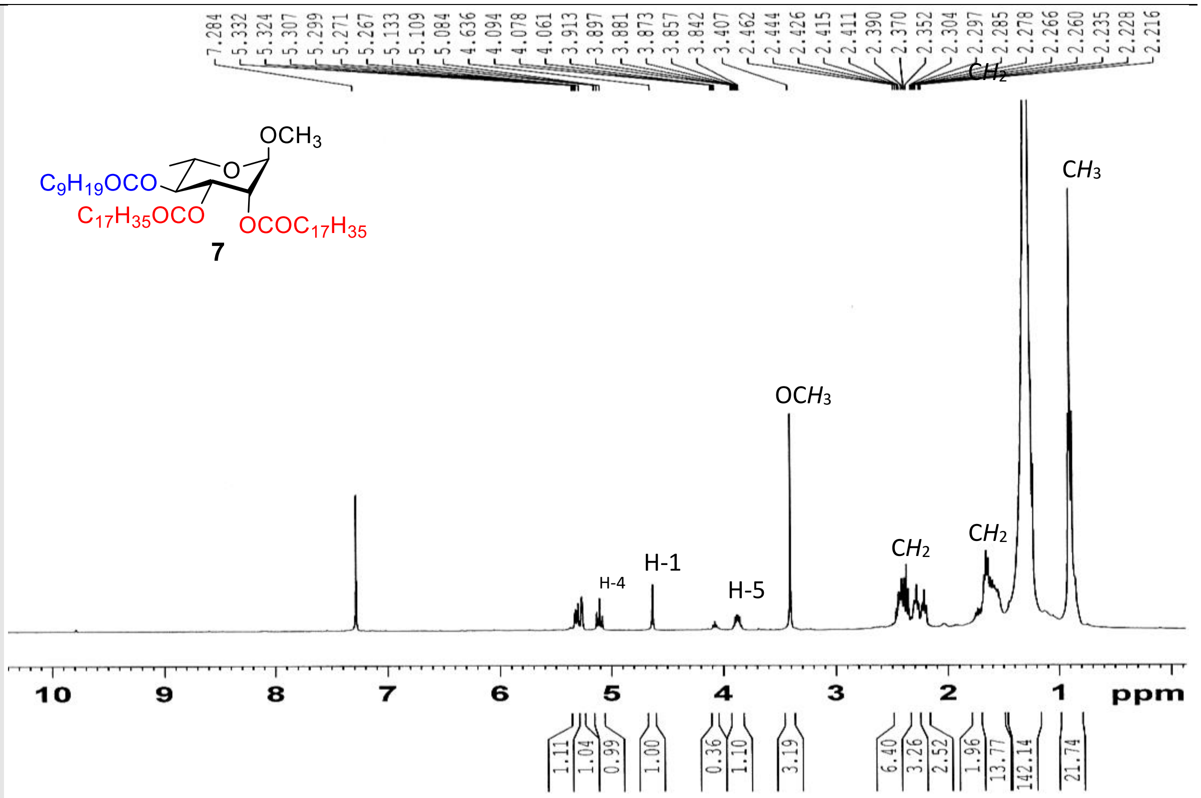

Figure S20. ^{1}H NMR (400 MHz, $CDCl_3$) spectrum of compound **7**.

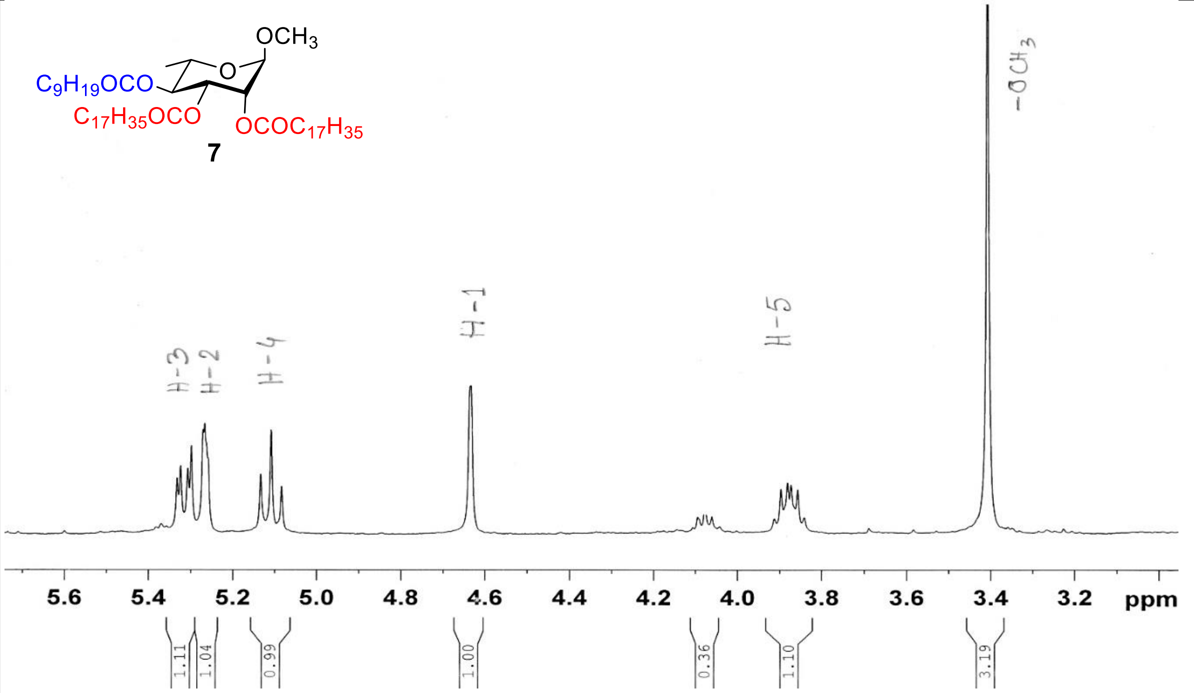

Figure S21. ^{1}H NMR (400 MHz, $CDCl_3$) spectrum (Expansion) of compound **7**.

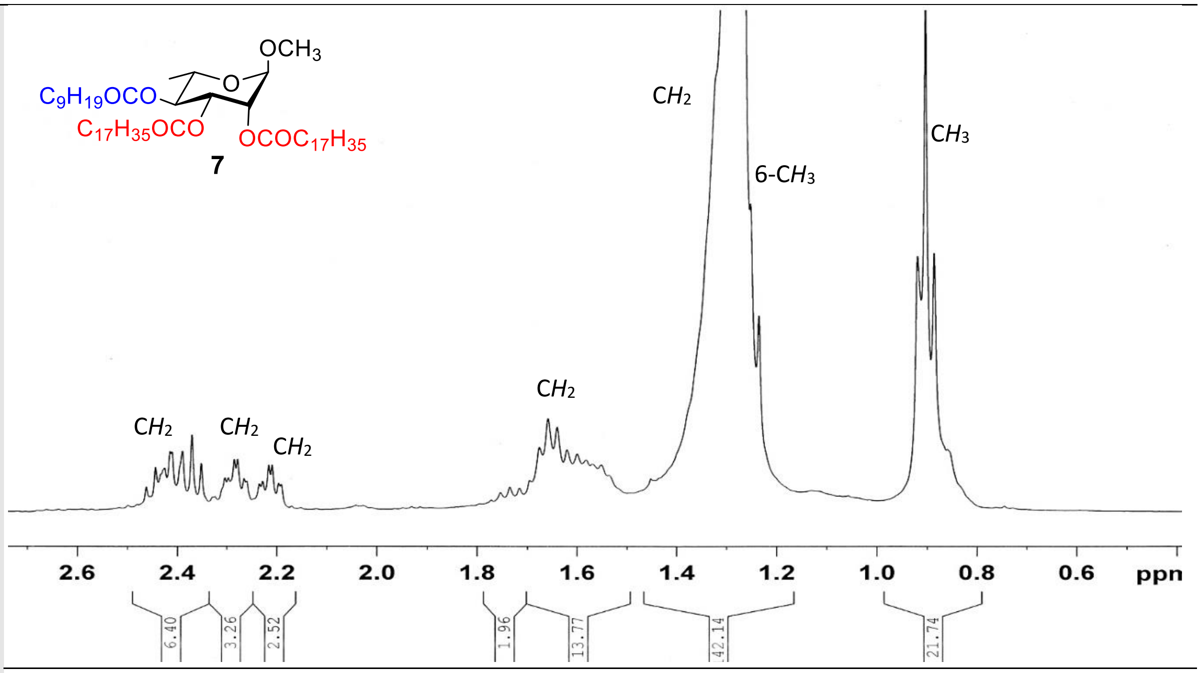

Figure S22. ^{1}H NMR (400 MHz, $CDCl_3$) spectrum (Expansion) of compound **7**.

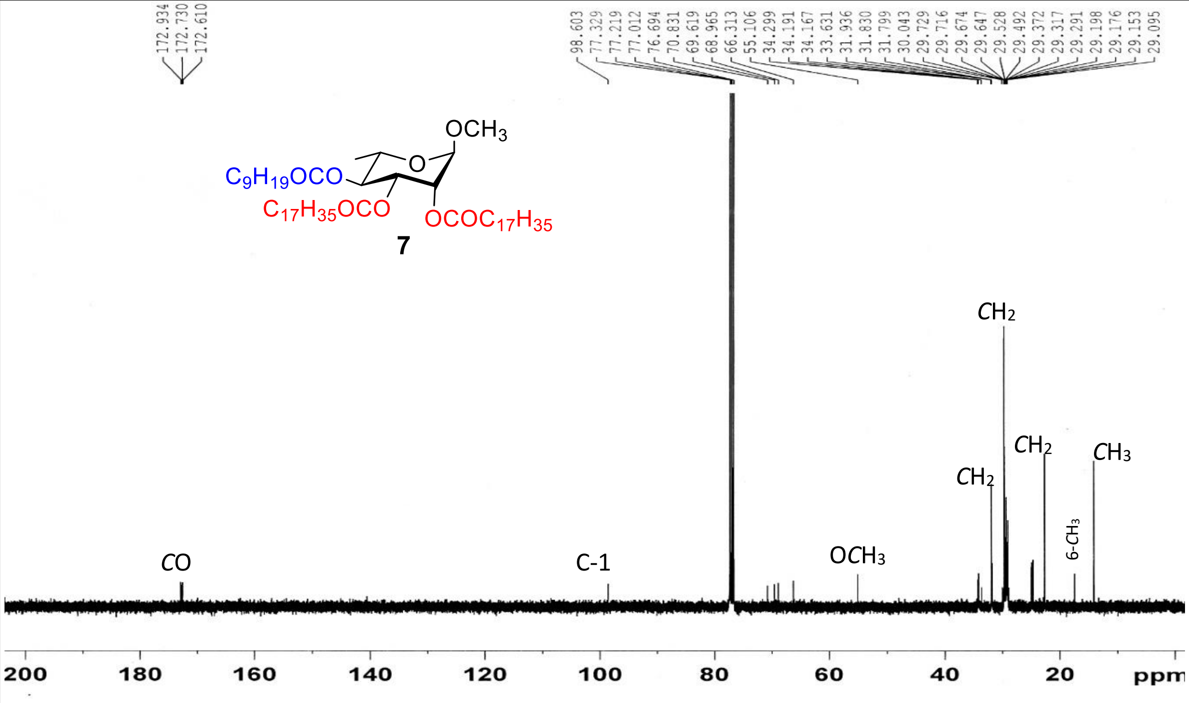

Figure S23. ^{13}C NMR (100 MHz, $CDCl_3$) spectrum of compound **7**.

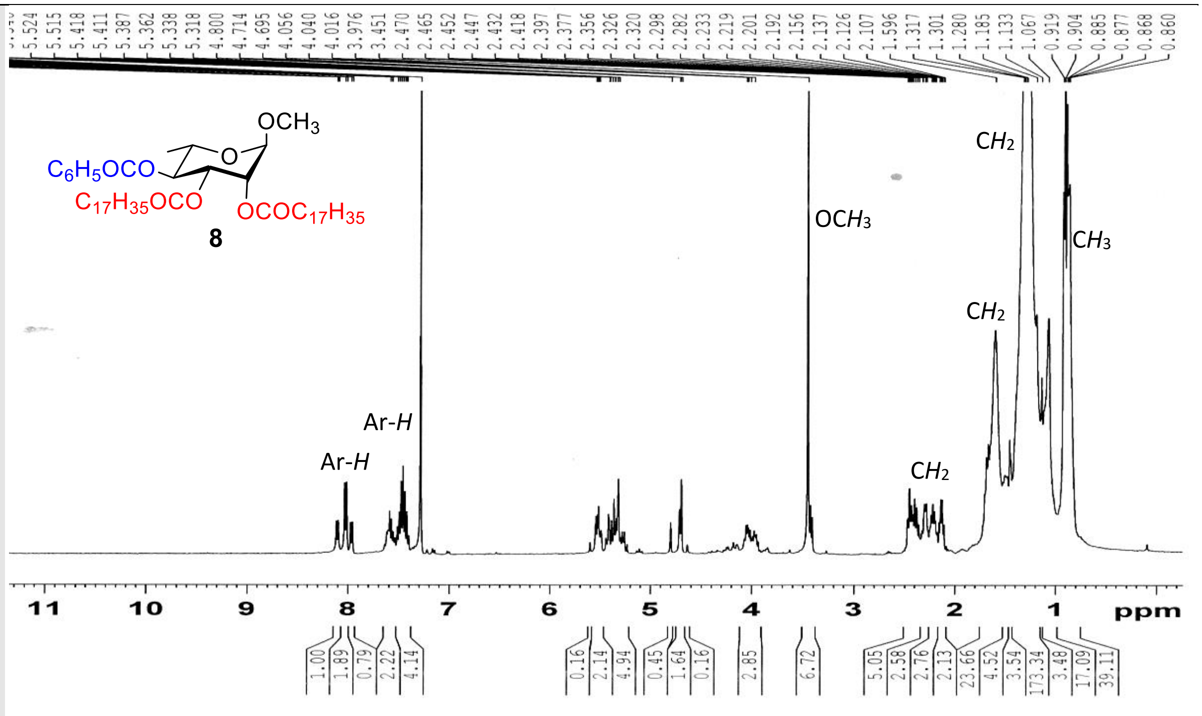

Figure S24. ^{1}H NMR (400 MHz, $CDCl_3$) spectrum of compound **8**.

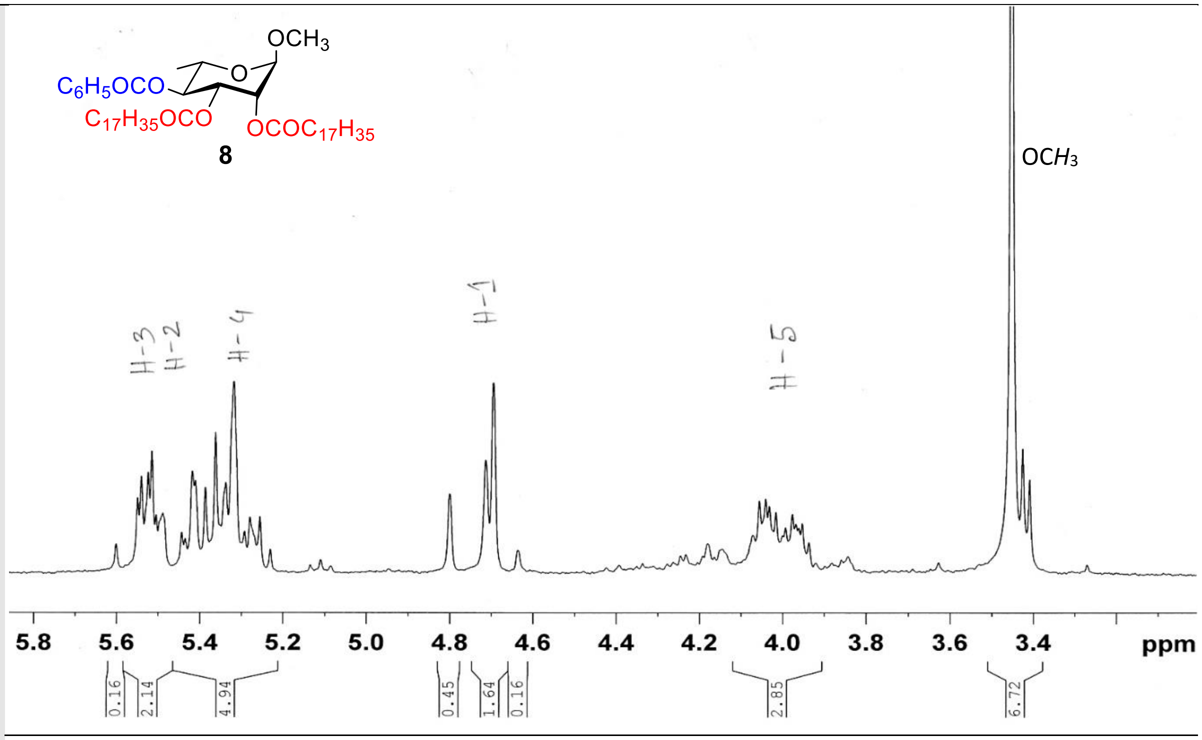

Figure S25. ^{1}H NMR (400 MHz, $CDCl_3$) spectrum (Expansion) of compound **8**.

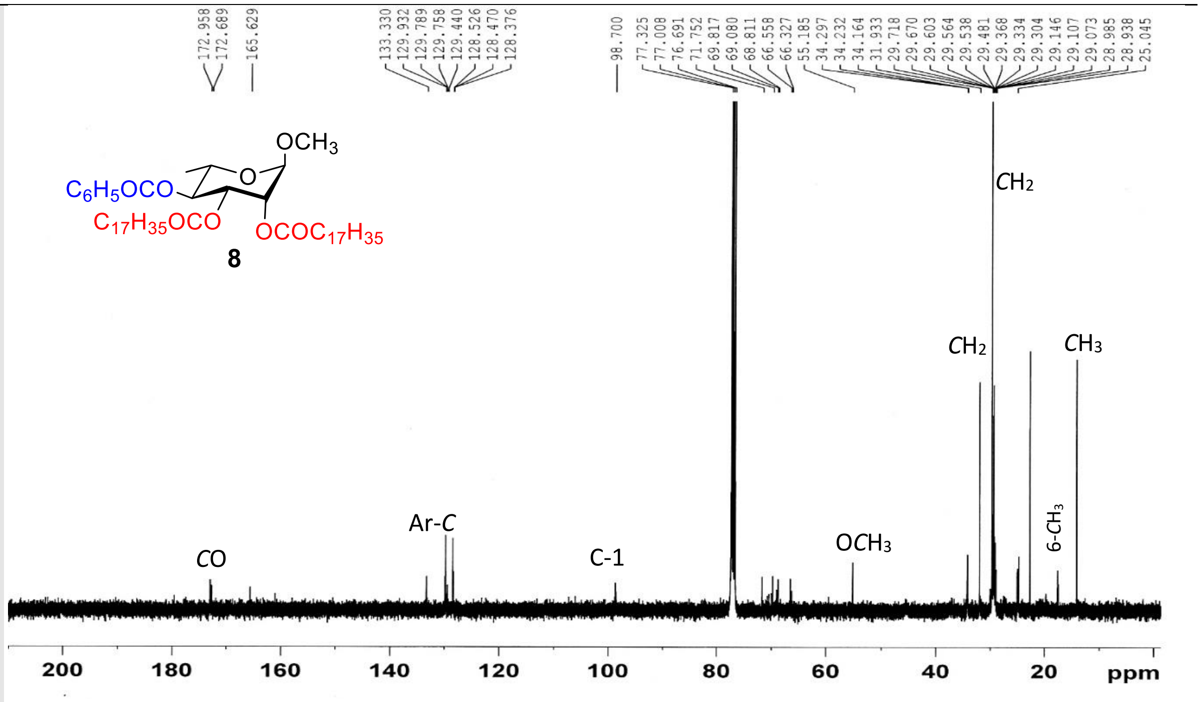

Figure S26. ^{13}C NMR (100 MHz, $CDCl_3$) spectrum of compound **8**.

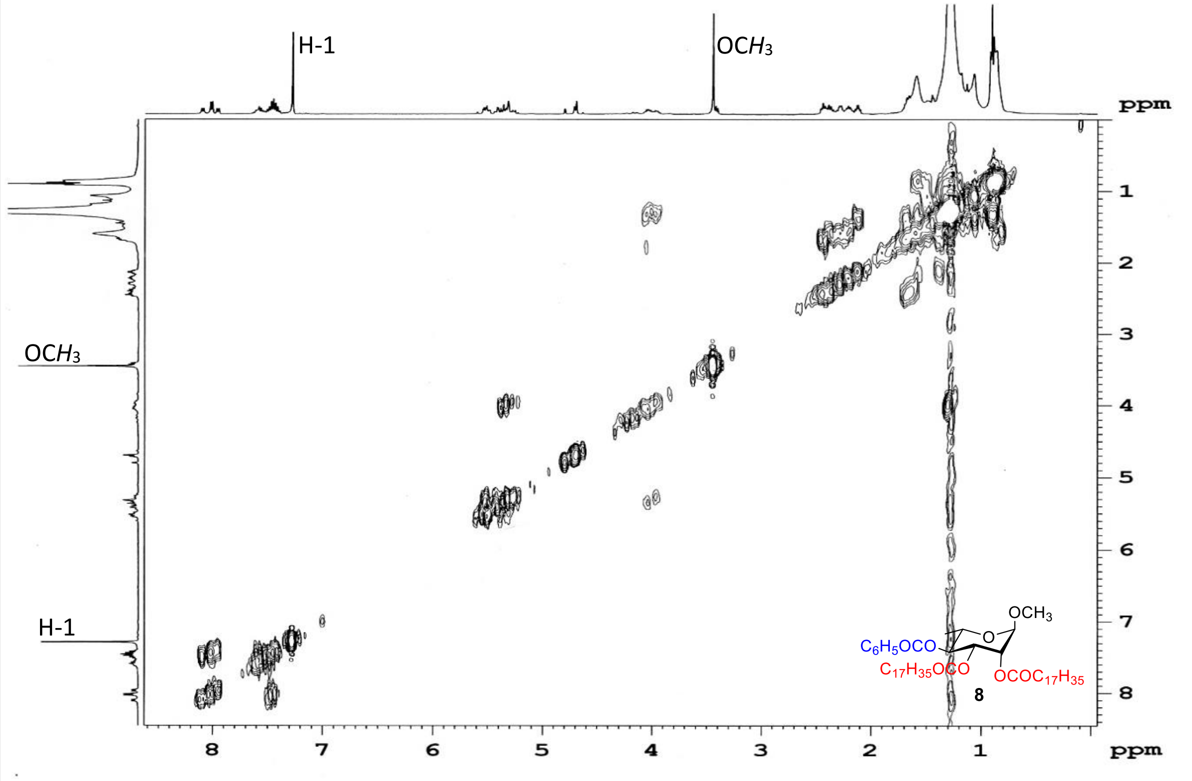

Figure S27. 2D COSY spectrum of compound **8**.

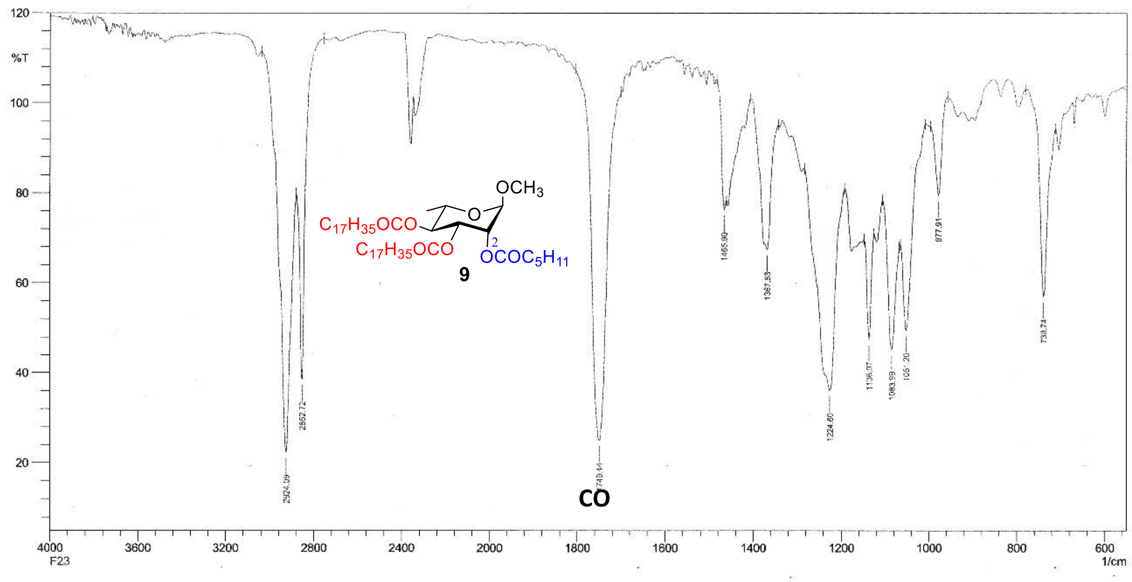

Figure S28. IR (neat) spectrum of compound **9**.

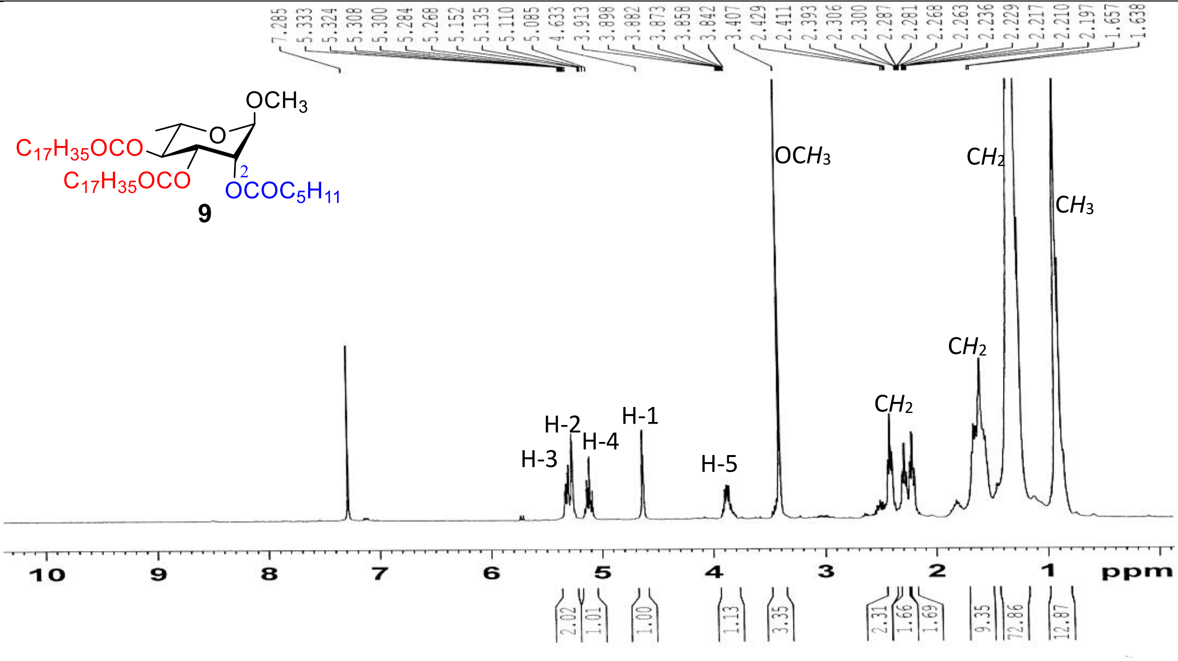

Figure S29. ^{1}H NMR (400 MHz, $CDCl_3$) spectrum of compound **9**.

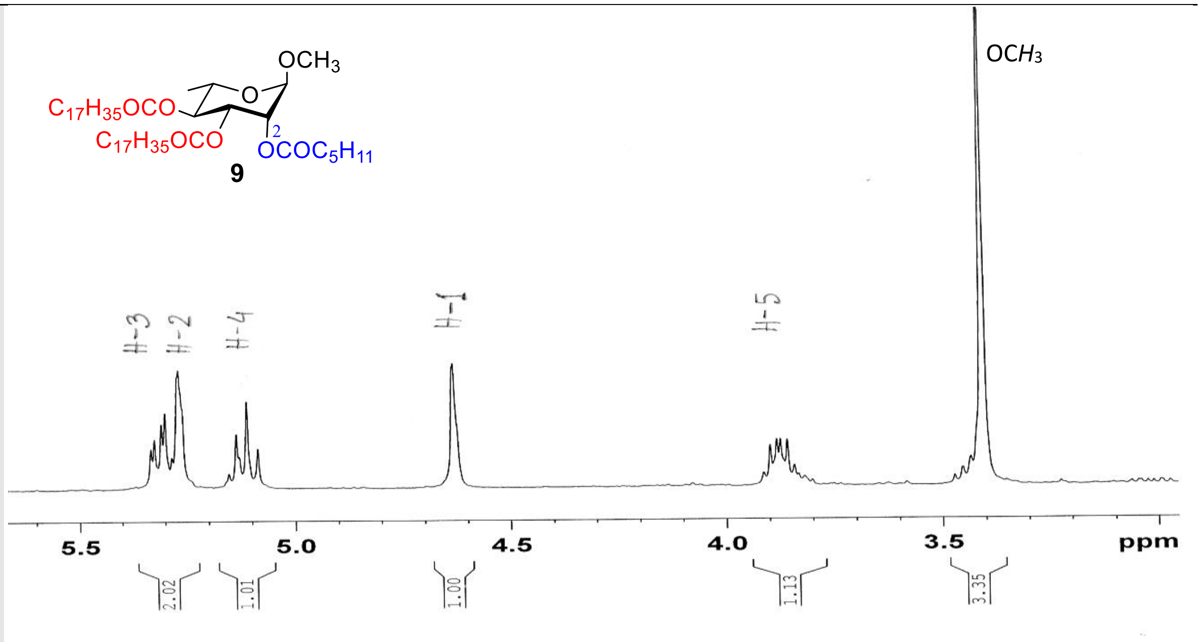

Figure S30. ^{1}H NMR (400 MHz, $CDCl_3$) spectrum (Expansion) of compound **9**.

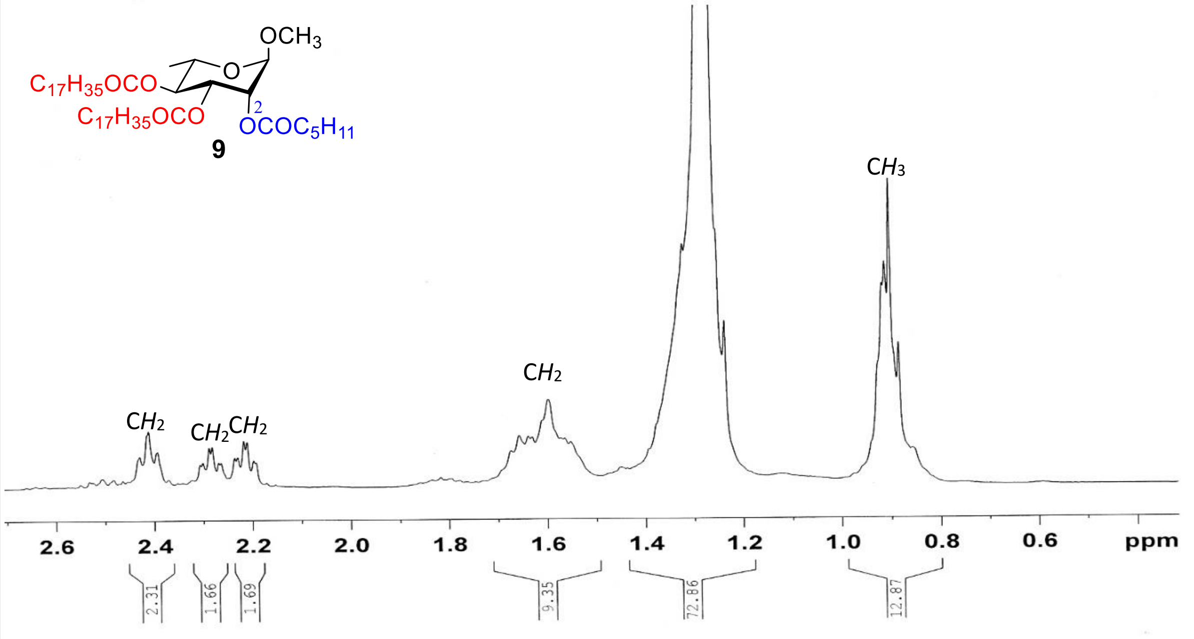

Figure S31. ^{1}H NMR (400 MHz, $CDCl_3$) spectrum (Expansion) of compound **9**.

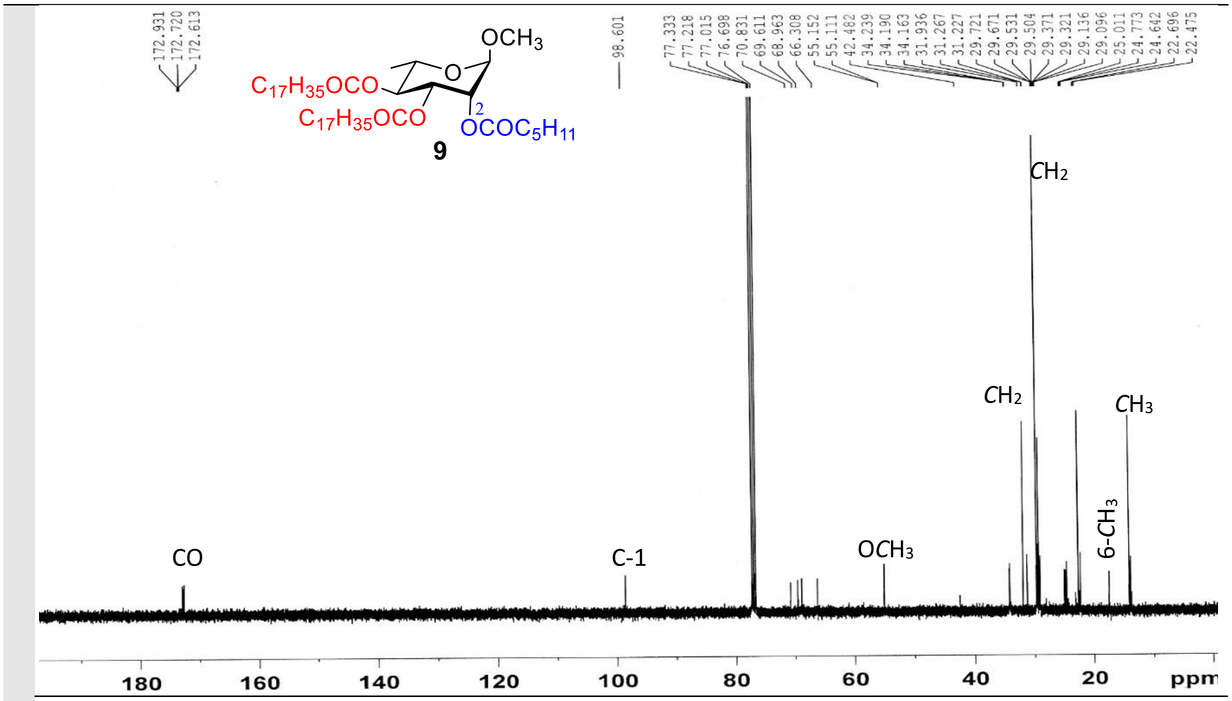

Figure S32. ^{13}C NMR (100 MHz, $CDCl_3$) spectrum of compound **9**.

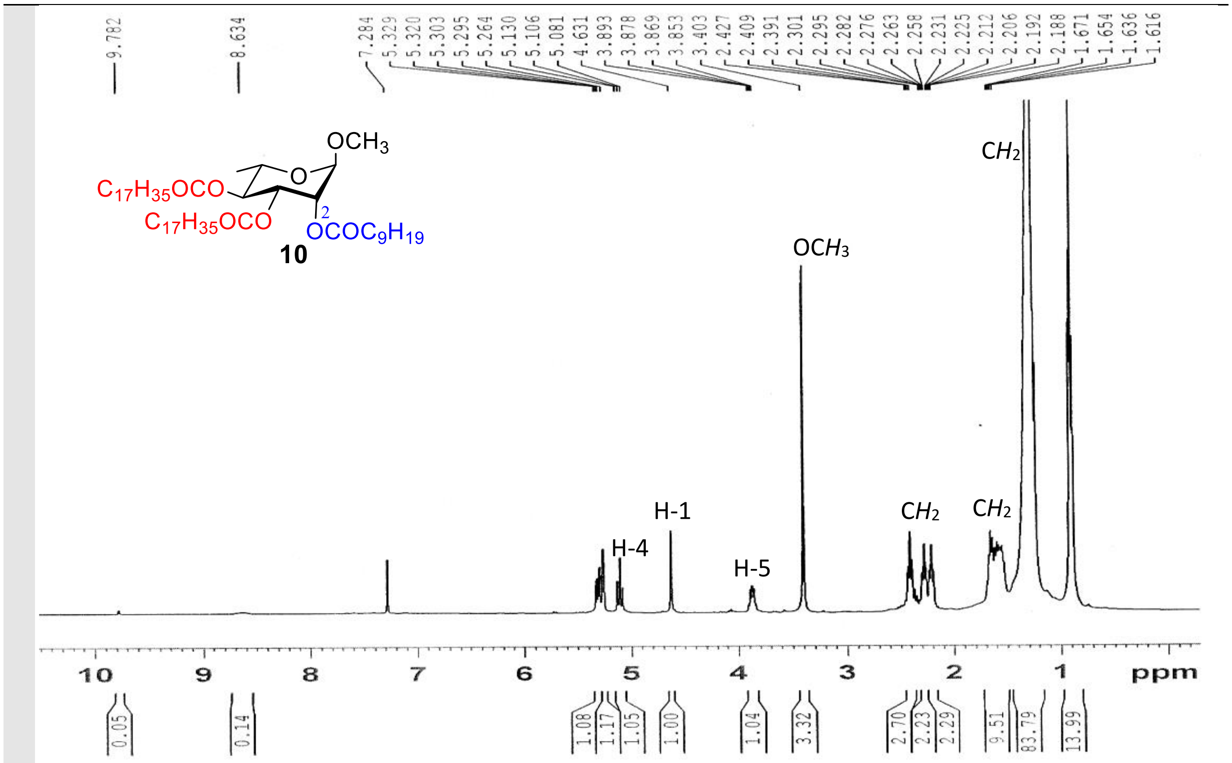

Figure S33. ^{1}H NMR (400 MHz, $CDCl_3$) spectrum of compound **10**.

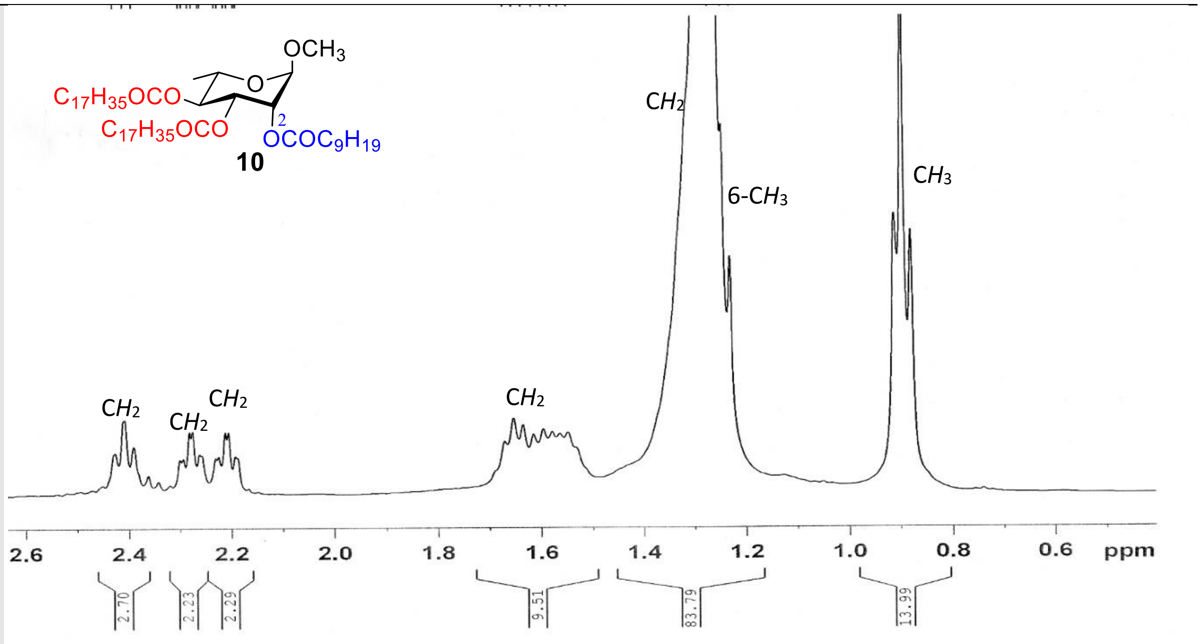

Figure S34. ^{1}H NMR (400 MHz, $CDCl_3$) spectrum (Expansion) of compound **10**.

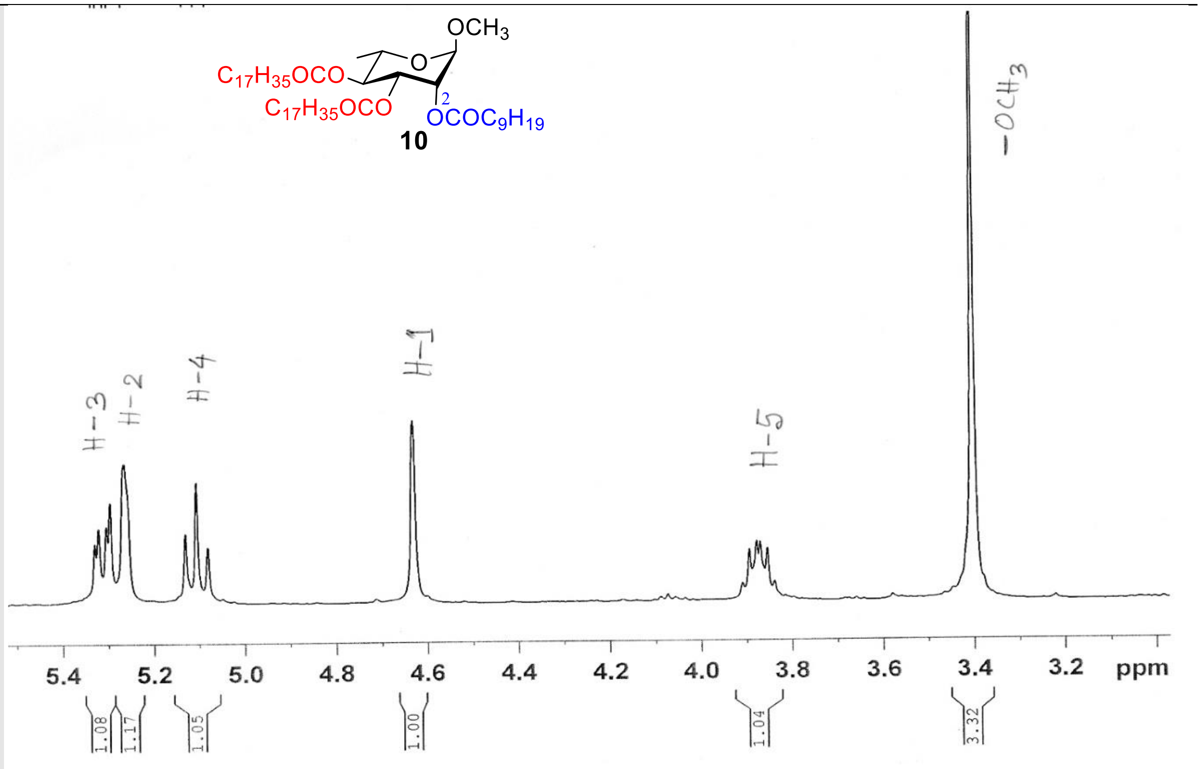

Figure S35. ^{1}H NMR (400 MHz, $CDCl_3$) spectrum (Expansion) of compound **10**.

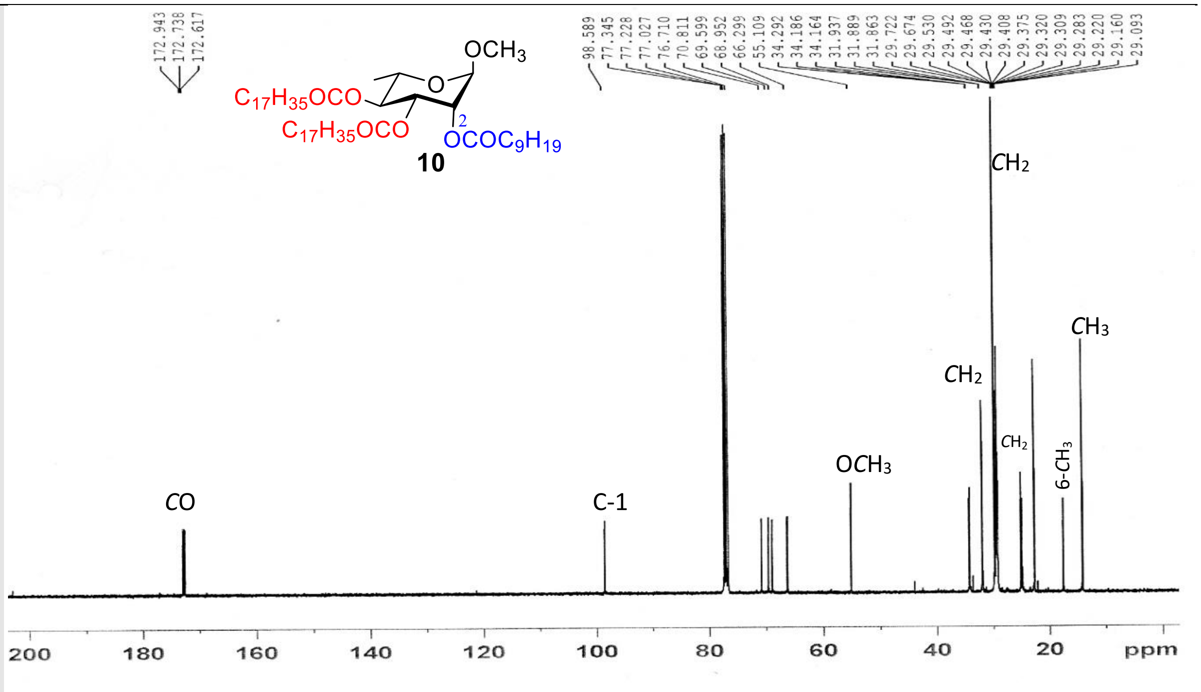

Figure S36. ^{13}C NMR (100 MHz, $CDCl_3$) spectrum of compound **10**.

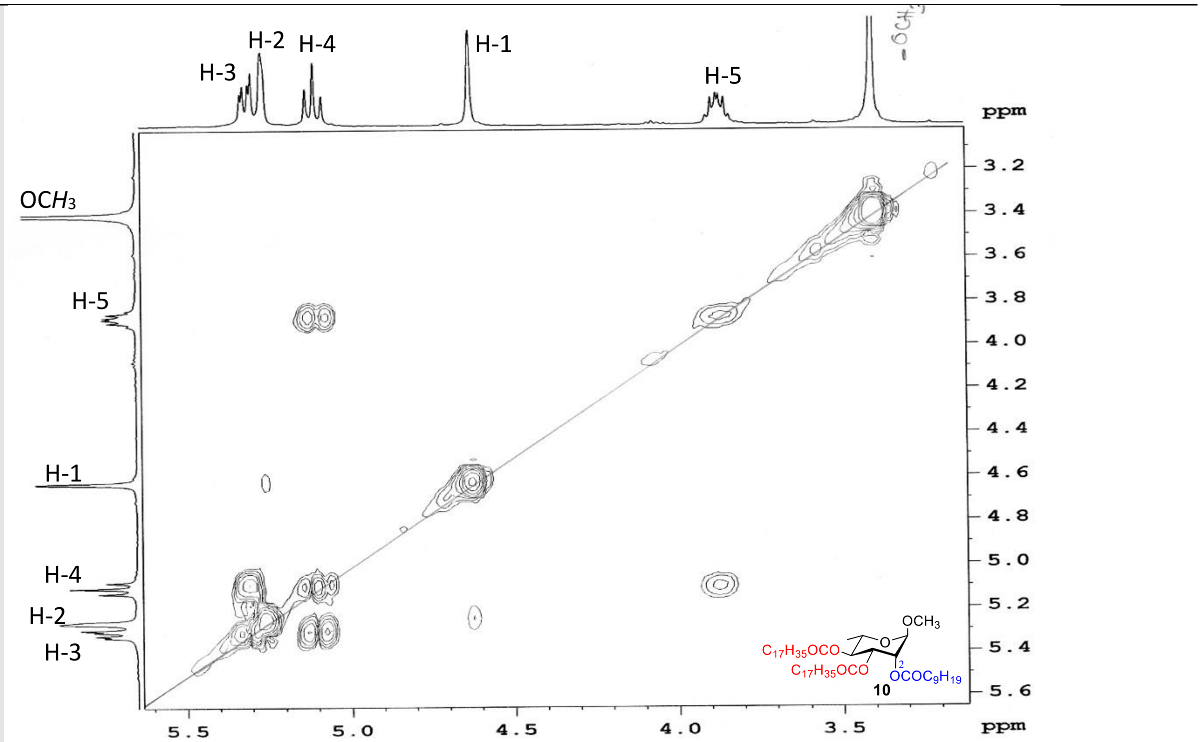

Figure S37. 2D COSY spectrum (Expansion) of compound **10**.

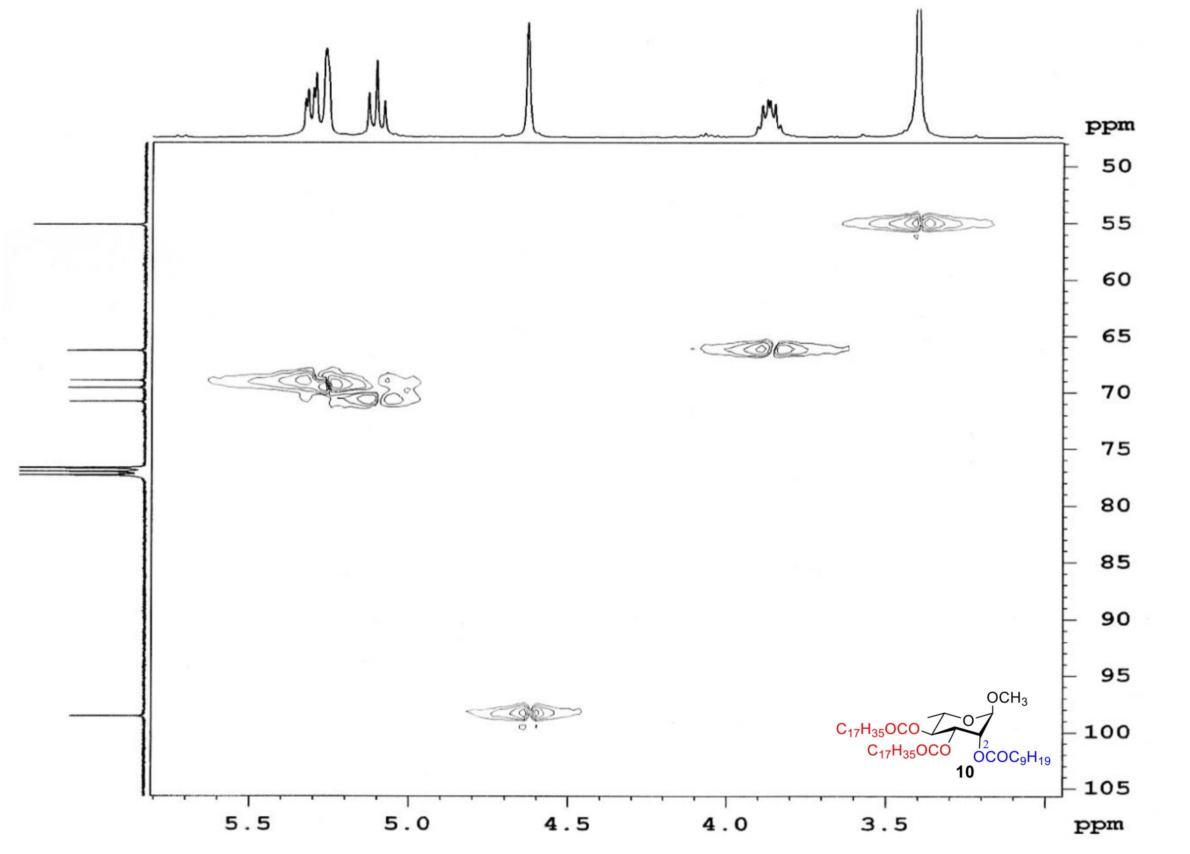

Figure S38. 2D HSQC spectrum (Expansion) of compound **10**.

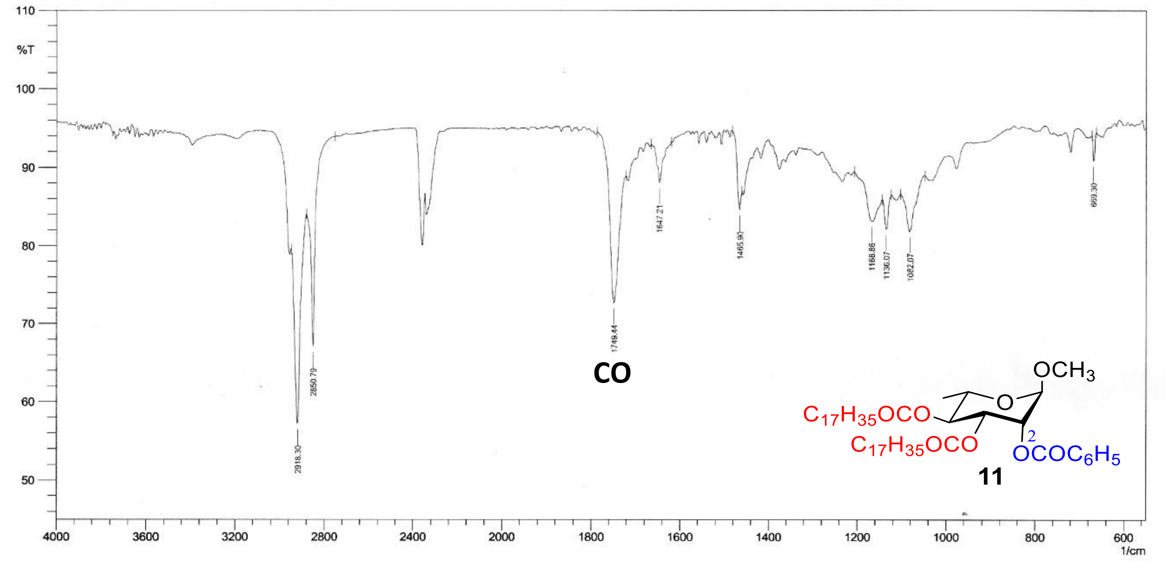

Figure S39. FT-IR (neat) spectrum of compound **11**.

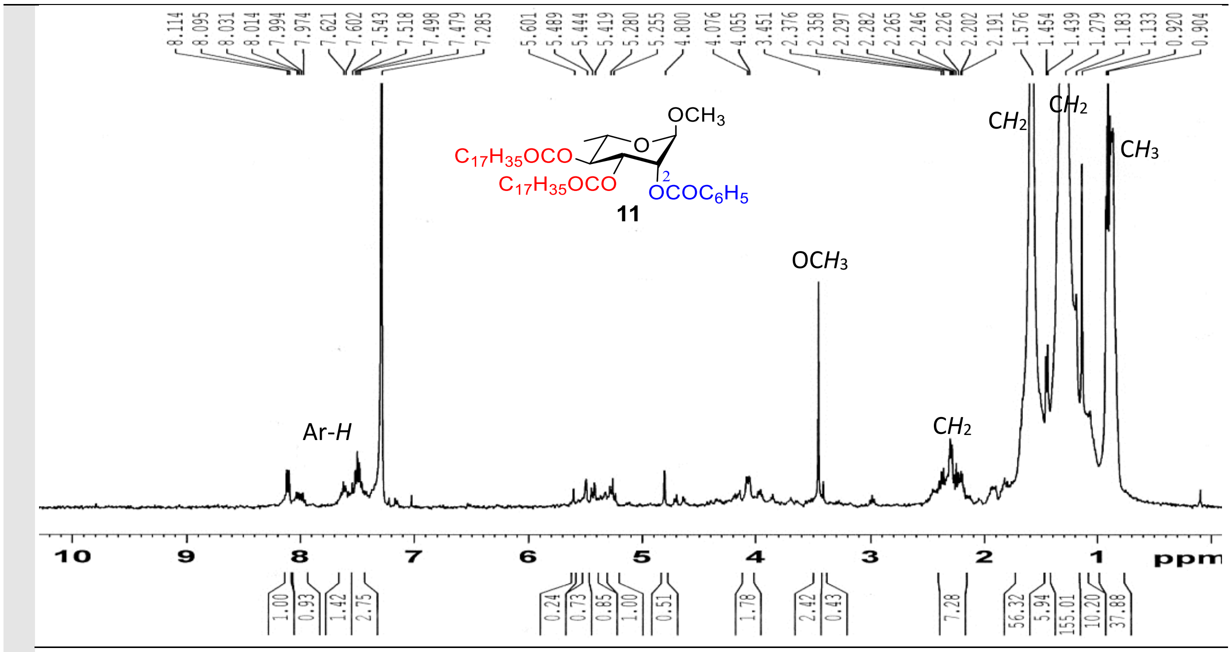

Figure S40. ^{1}H NMR (400 MHz, $CDCl_3$) spectrum of compound **11**.
